# IgE-binding residues analysis of the house dust mite allergen Der p 23

**DOI:** 10.1038/s41598-020-79820-y

**Published:** 2021-01-13

**Authors:** Sze Lei Pang, Sri Anusha Matta, Yang Yie Sio, Yu Ting Ng, Yee-How Say, Chyan Leong Ng, Fook Tim Chew

**Affiliations:** 1 Present Address: Department of Biological Sciences, National University of Singapore, 14 Science Drive 4, Singapore, 117543 Singapore; 2 Institute of Systems Biology, Universiti Kebangsaan Malaysia, 43600 UKM Bangi, Selangor Malaysia; 3 Department of Biomedical Science, Faculty of Science, Universiti Tunku Abdul Rahman (UTAR) Perak Campus, 31900 Kampar, Perak Malaysia

**Keywords:** Immunology, Structural biology

## Abstract

House dust mites (HDMs) are one of the major causes of allergies in the world. The group 23 allergen, Der p 23, from *Dermatophagoides pteronyssinus*, is a major allergen amongst HDM-sensitized individuals. This study aims to determine the specific immunoglobulin E (sIgE) binding frequency and IgE-binding residues of recombinant Der p 23 (rDer p 23) allergen amongst a cohort of consecutive atopic individuals in a tropical region. We performed site-directed mutagenesis and carried out immuno-dot blot assays using 65 atopic sera. The immuno-dot blot assays results indicated that the two residues K44 and E46 which are located at the N-terminal region are the major IgE-binding residues. The rDerp-23 sIgE titers are strongly correlated to the number of IgE-binding residues for rDer p 23 (*P* < 0.001). Atopic individuals who were only sensitized to HDM have a significantly higher number of IgE-binding residues than the individuals who were polysensitized to HDM and other crude allergens (*P* < 0.05). Individuals with allergic multimorbidity and moderate-to-severe allergic rhinitis also have a higher number of IgE-binding residues compared to those with single allergic disease and mild allergic rhinitis. The results prompt us to hypothesize that the individuals who have a higher number of IgE-binding residues may face a bigger challenge to be treated through immunotherapy due to the complexity in designing an effective hypoallergen with a high number of IgE-binding residues. We propose that the development of a refined molecular diagnostic assay, which includes alanine substitution of surface-exposed residues could be a more precise diagnostic strategy to identify all the IgE-binding residues of a major allergen for an atopic individual and the development could be another new dimension in allergy diagnosis and allergen immunotherapy treatment.

## Introduction

House dust mites (HDMs) have been described as the major source of indoor allergens capable of sensitizing and triggering allergic symptoms in genetically predisposed/atopic individuals. Allergic diseases triggered by HDM allergens include asthma (AS), allergic rhinitis (AR), and atopic dermatitis (AD). The main species of HDMs are *D. pteronyssinus*, *D. farinae*, *Euroglyphus maynei*, and *Blomia tropicalis*. The prevalence of HDM species varies depending on the geographical location^1–4^. *D. pteronyssinus* and *D. farinae* have been shown as the common HDMs worldwide. Whereas, *B. tropicalis* is the predominant mite species in tropical and subtropical regions, and in some areas, it coexists with *D. pteronyssinus*.

The incorporation of both molecular biology technique and high throughput omics approaches in allergy research allows the identification of more than 30 HDM allergens (including isoallergens and variants) (www.allergen.org). Recently, group 23 allergen including Der p 23 and Der f 23 were identified in *D. pteronyssinus* and *D. farinae*, respectively^5–9^. Der p 23 was identified as a major *D. pteronyssinus* allergen^5,6^. Der p 23 was recognized in > 70% of HDM-allergic patients, and the Der p 23-sIgE levels of the patients were comparable to the two major HDM allergens, Der p 1 and Der p 2^5^. The IgE-binding residues of Der p 23 among population in tropical regions, however, remains to be identified.

Studies have focused on identifying the IgE-binding epitope of allergens to improve the diagnosis and immunotherapy of HDM allergy^10–16^. The correlation between IgE titer and IgE-repertoire complexity was demonstrated for HDM allergy^17^. Sera with increased Der p 2-sIgE titers were shown to correlate with the increased complexity of the IgE repertoires^17^. A comprehensive IgE epitope screening using all the surface-exposed residues of rDer f 21 (group 21 allergen) from *D. farinae* has highlighted the positive correlation between the number of IgE-binding residues and rDer f 21-sIgE level^15^. While IgE antibody binding to an increasing number of IgE-binding residues has been associated with sIgE level, the information regarding the clinical significance of the number of IgE-binding residues remained to be identified.

This study aims to identify the IgE-binding residues of rDer p 23 allergen and the correlation between the number of IgE-binding residues and rDerp23-sIgE titers of atopic individuals. Furthermore, the correlation of the number of IgE-binding residues with monosensitization/polysensitization to HDM, allergic multimorbidity, and the severity of AR was also analyzed.

## Methods

### Study participants

Volunteers were recruited at Universiti Tunku Abdul Rahman for the study on the genetics of obesity and allergic diseases. Participants were classified as individuals with AS, AR, AD or healthy using a questionnaire that designed based on the Allergic Rhinitis Impact on Asthma (ARIA)^18,19^ and the International Study of Asthma and Allergies in Childhood (ISAAC)^20^ guidelines. The validated questionnaire includes questions on demographics, anthropometric measurements, medical history, and environmental exposure. The details of diagnostic criteria for AS, AR, and AD are specified in the supporting information.

### Skin prick test (SPT)

The participants were also subjected to a SPT using common allergens in Singapore and Malaysia, based on previous reported studies^3,21–23^. The SPT result for each tested allergen was defined as positive if the mean wheal diameter was 3 mm or larger than the negative control (saline). Histamine (1 mg/mL; Diater, Spain) was used as positive control. The raw materials for extract preparation were cultured in-house as described^24^, except for *D. pteronyssinus* and *B. tropicalis* mites (purchased from Siriraj Dust Mite Center for Services and Research, Mahidol University, Thailand). The SPT panel included *D. pteronyssinus*, *B. tropicalis*, *E. guineensis*, and *C. lunata* extracts. All the four extracts were prepared at 0.1 mg/mL in 50% glycerol.

### Cloning, expression, and purification of rDer p 23

Recombinant Der p 23 was prepared as described in the supplementary information (see supplementary method). Comparison of Der p 23 protein sequences are shown in Supplementary Fig. S1.

### Mass spectrometric analysis and protein identification of rDer p 23

Protein identification of rDer p 23 was conducted using mass spectrometry (see Supplementary Method).

### Generation of rDer p 23 mutants

Recombinant Der p 23 mutants were generated and produced as described in the supplementary information (see Supplementary Method).

### Screening of sera of atopic individuals by immuno-dot blot assay

One microgram of *D. pteronyssinus* crude extract and rDer p 23 proteins (wild type and 17 mutants) were dotted in duplicate onto nitrocellulose membrane together with a serial dilution of IgE standard (National Institute for Biological Standards, United Kingdom; two-fold serial dilution from 1000 to 3.91 IU/mL) as a standard curve (Supplementary Fig. S9). 1 µg of bovine serum albumin and 1 µL of protein buffer as negative controls. The membrane was air-dried, blocked with PBS-T 0.1% (1 X PBS with 0.1% Tween 20) and then incubated overnight with sera (1:4 in PBS) at 277 K. After washing, the membrane was incubated with anti-human IgE antibodies conjugated with alkaline phosphatase (1:1000 in PBS). The alkaline phosphatase activity was then detected by the addition of nitro-blue tetrazolium/5-bromo-4-chloro-3-indolyl-phosphate chromogenic substrate for 10 min. Subsequently, the developed membranes were photographed using gel documentation camera (Syngene) and were analyzed using GeneTools software (Syngene). Spot intensities were normalized by subtracting the local background intensity. Intensities that are larger than 40.22 arbitrary units (equivalent to 2 standard deviations above the mean negative sera responses) were considered positive. This is equivalent to approximately 4 IU/mL, calculated using the logarithmic equation from the IgE standard curve (Supplementary Fig. S9). The assay performed in this study has been previously used and validated in other publications^10,12,14–16,25^.

### Statistical analysis

GraphPad Prism 7 was used to generate graphs. GraphPad Prism 7 and IBM SPSS Statistics version 25 software (IBM Corp., Armonk, NY) were used for statistical analysis. The tests included: Spearman rank-order correlation coefficients for correlation; chi-square test or Fisher’s exact test for comparing frequencies of two possible outcomes. All comparisons were two-tailed; Mann–Whitney test for comparing the median of two possible outcomes. *P* values less than 0.05 were considered as statistically significant.

### Ethics approval and consent to participate

Human subjects recruited in this study approved by Scientific and Ethical Review Committee (SERC) in the Universiti Tunku Abdul Rahman (UTAR), Malaysia (UTAR-SERC, Ref code: U/SERC/36/2015 and U/SERC/03/2016). All recruitment procedures were performed in concordance with the Helsinki Declaration. Written informed consents were obtained from all the participants.

## Results

### Expression, purification, and biochemical characterization of rDer p 23 protein

The Der p 23 gene sequence that codes for amino acid residues 44–90 was synthesized, by referring to the reported crystal structure of Der p 23 (PDB code: 4ZCE)^6^. Exclusion of the N-terminal 43 residues was due to (1) the putative signal peptide for the first 21 amino acid residues, and (2) the N-terminal residues 21–43 were reported to be largely unstructured^5^ (Supplementary Fig. S1). In addition, previous study also showed that the residues 22–52 did not affect IgE recognition^7^.

The C-terminal 6 × His-tagged rDer p 23 protein was expressed in *E. coli* strain Rosetta-gami (DE3) and purified by a two-step procedure involving Ni-NTA affinity chromatography and size-exclusion chromatography (SEC). The SEC profile shows that Der p 23 protein was eluted at the retention volume of 238.6 mL, with an estimated molecular weight of 3.57 kDa, between 17 kDa and 1.35 kDa. The 15% SDS-PAGE analysis indicated the presence of rDer p 23 protein at ~ 10 kDa (Supplementary Fig. S2). The calculated molecular weight of rDer p 23 from the deduced amino acid is 6.85 kDa. Mass spectrometry using TripleTOF 5600 (AB Sciex) confirmed the identity of the purified protein to be rDer p 23 (Table S1), with 35.6% of the peptide sequence matching that of Der p 23 (Accession number: ACB46292) (Supplementary Fig. S3 and Table S2).

### Site-directed mutagenesis and purification of rDer p 23 mutants

**Figure 1:**
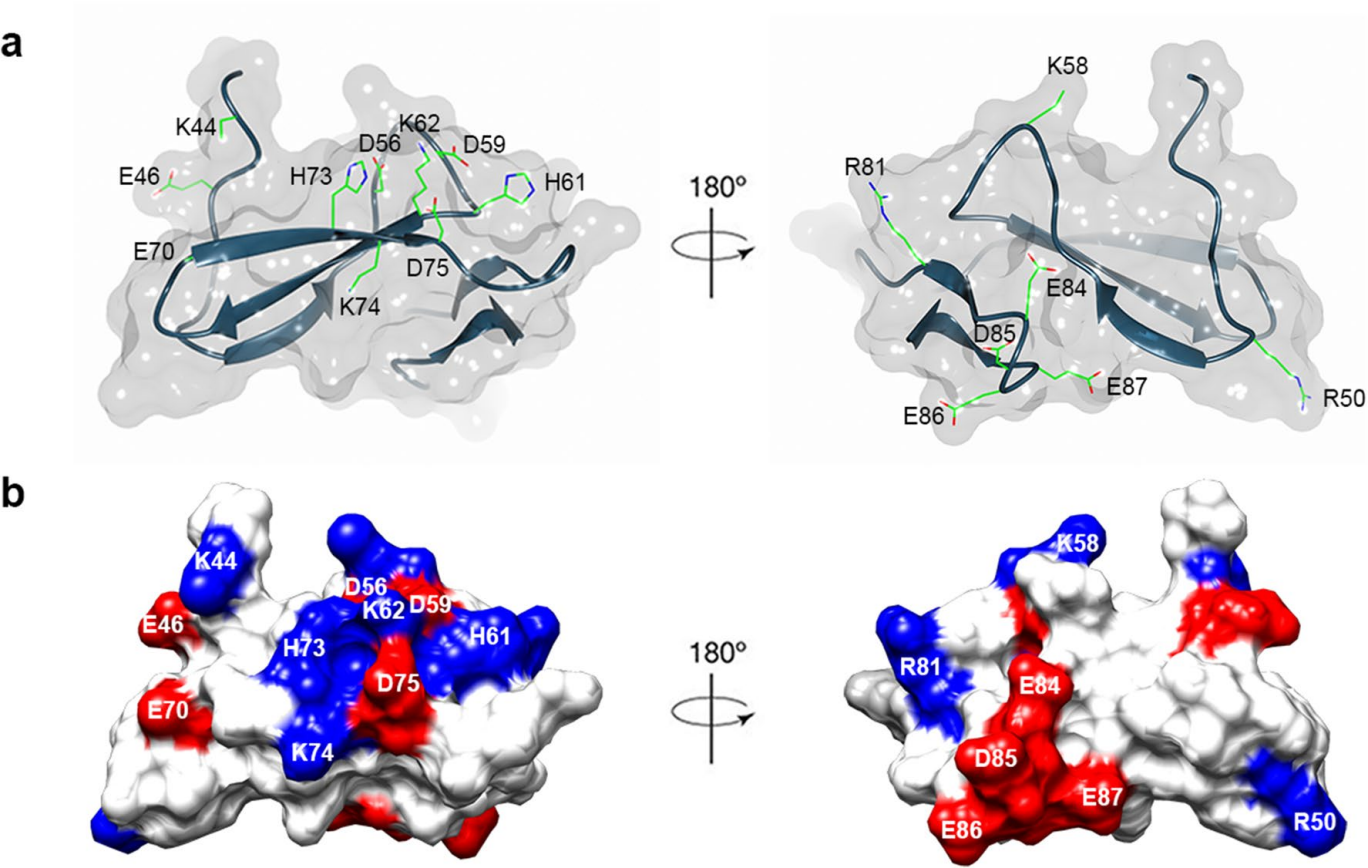
The 17 solvent-accessible, polar, and charged amino acid residues selected for mutagenesis. (**a**) All the selected residues were mapped and shown as cylinder models on the ribbon diagram of rDer p 23 chain A. (**b**) Surface diagram of rDer p 23 with selected residues highlighted. Positively charged and negatively charged residues are colored in blue and red, respectively. Figures were generated using the program CCP4mg^36^ and the program Chimera^37^, respectively.

The solvent accessibility of residues was determined using AREAIMOL from CCP4 suite^26^ based on the crystal structure of Der p 23 dimer (chain A and B) (PDB code: 4ZCE)^6^. The ratio of solvent-accessible surface area (ASA) to the calculated Gly-X-Gly (GXG) tripeptide value for all the residues was obtained. Previous IgE epitope mapping of dust mite allergens from groups 5, 13, and 21 found that the major IgE-binding epitopes consist of polar and charged residues^10,12,15,16^. Hence, we identified all solvent-accessible, polar and charged residues (17 in total) on the surface of Der p 23, and mutated these residues to alanine to produce a set of 17 single-residue mutants. All the mutants were mapped on the rDer p 23 crystal structure as shown in Fig. 1.

A total of 17 mutant gene sequences were synthesized and cloned into pET-28b(+) expression vector. Same with wild type rDer p 23, all the mutants were expressed in *E. coli* strain Rosetta-gami (DE3) and purified by nickel-nitrilotriacetic acid (Ni-NTA) affinity chromatography. As shown in Supplementary Fig. S4, all the 17 mutants were purified successfully.

### Secondary structure analyses of purified rDer p 23 and mutants

To ensure that the loss of IgE binding were due to the loss of IgE-binding residues instead of the overall structure of the protein, we have performed far-UV circular dichroism (CD) analysis for four selected mutants, including K44A, E46A, E70A, and H73A. The secondary structure analysis by far-UV CD showed that wild type rDer p 23 is mainly a random coil structure, with the characteristic minima at ~ 195 nm, similar with the reported CD spectrum of Der p 23 protein^5,9^. The far-UV CD spectra indicated that all the four mutants retained their predominant random coil secondary structure, closely resembling the CD spectrum of the wild type rDer p 23 (Supplementary Fig. S5). The CAPITO analysis of spectra also showed the similar secondary structures in the wild type rDer p 23 and its mutants (Table S3)^27^.

### Characteristics of participants and the frequency of IgE binding to rDer p 23

**Figure 2:**
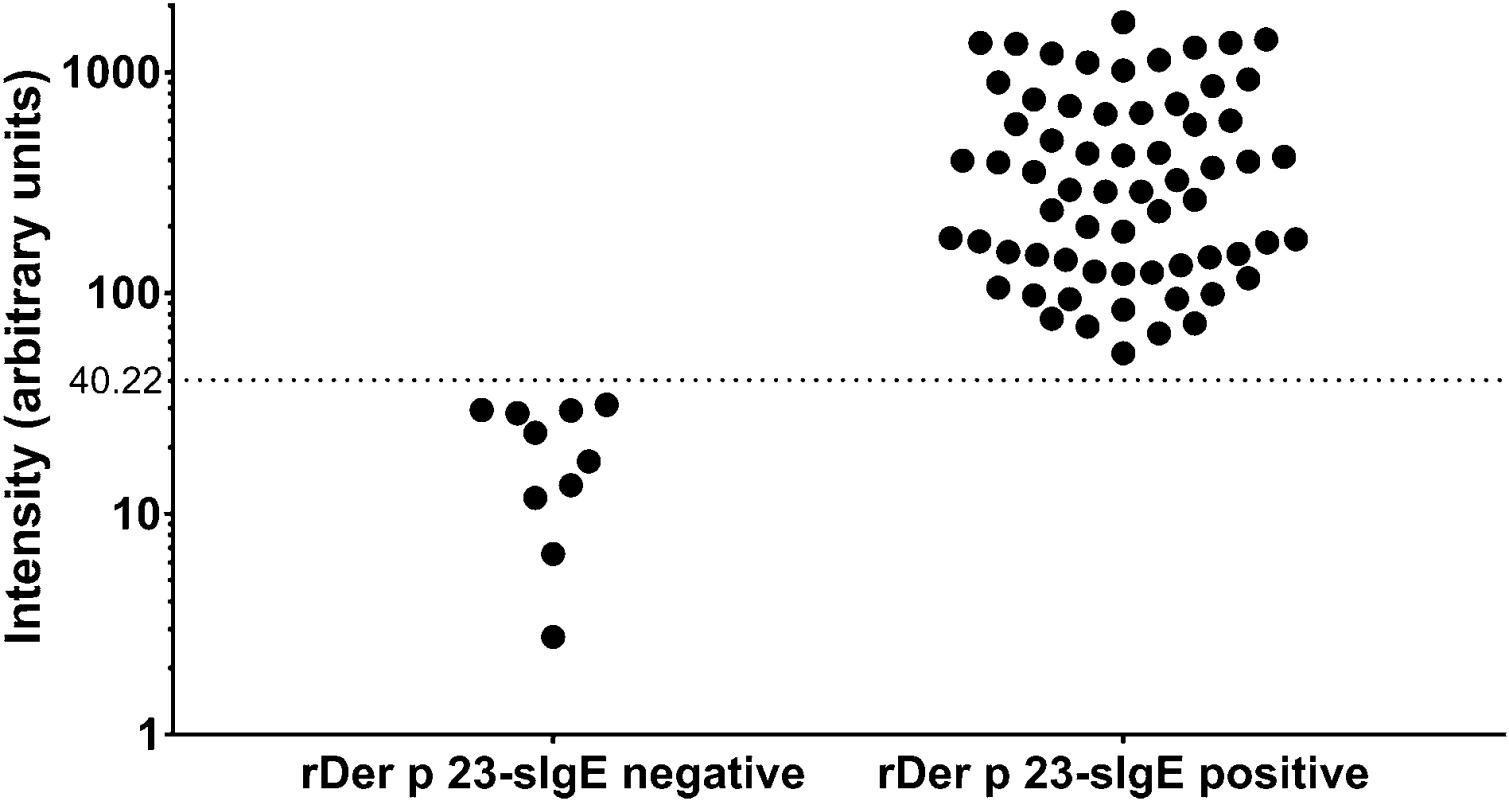
Intensity (arbitrary unit (a.u.)) of rDer p 23-specific immunoglobulin E (sIgE)-negative and sIgE-positive individuals. Individuals with intensity < 40.22 a.u. were defined as rDer p 23-sIgE negative (n = 10). The 65 rDer p 23-sIgE positive individuals have the intensity between and equal to 53.25 a.u. to 1682.41 a.u.

A total of 75 participants, 18–27 years old were included and classified into rDer p 23-sIgE positive and rDer p 23-sIgE negative groups based on the results of SPT and immuno-dot blot assay. The rDer p 23-sIgE positive group (intensity range 53.25–1682.41 a.u.) consisted of 65 individuals who were sensitized to both crude *D. pteronyssinus* and rDer p 23 allergens. Whereas, the 10 individuals who were non-sensitized to rDer p 23 with intensity reading less than 40.22 (intensity range 17.30–29.53 a.u.) were classified as rDer p 23-sIgE negative group (Fig. 2), which used as the negative control group in this study.

Comparisons between the groups revealed a similar proportion of gender and SPT sensitization to common inhalant allergens between rDer p 23-sIgE positive and rDer p 23-sIgE negative groups. The ratio of male to female participants within both groups is approximately 60 : 40. Overall SPT sensitization to another HDM allergen *B. tropicalis* was higher than *C. lunata* and *E. guinensis* for both the groups (Table 1).

**Table 1:** Characteristics of participants included in the study.

Characteristics	rDer p 23 sIgE-positive	rDer p 23 sIgE-negative
**N**	65	10
**Age (years)**	19.6 (range 18–27)	19.7 (range 18–23)
**Gender**		
Male	41 (63%)	6 (60%)
Female	24 (37%)	4 (40%)
**Skin prick test positive**		
*Dermatophagoides pteronyssinus*	65 (100%)	10 (100%)
*Blomia tropicalis*	56 (86%)	7 (70%)
*Curvularia* spp.	2 (3%)	1 (10%)
*Elaeis guineensis*	5 (8%)	2 (20%)
**Asthma**	12 (18%)	1 (10%)
Wheezing with exercise	2 (3%)	0
Nocturnal cough	3 (5%)	0
**Allergic rhinitis**	39 (60%)	3 (30%)
Symptoms		
Itchy nose	28 (43%)	3 (30%)
≥ 4 days/week	6 (9%)	0
≥ 4 weeks consecutively	4 (6%)	0
Sneezing	33 (51%)	2 (20%)
≥ 4 days/week	26 (40%)	0
≥ 4 weeks consecutively	4 (62%)	0
Runny nose	29 (45%)	3 (30%)
≥ 4 days/week	5 (8%)	0
≥ 4 weeks consecutively	4 (6%)	0
Nose blockage	31 (48%)	3 (30%)
≥ 4 days/week	3 (5%)	0
≥ 4 weeks consecutively	3 (5%)	0
Snore	7 (11%)	1 (10%)
≥ 4 days/week	0	1 (10%)
≥ 4 weeks consecutively	0	1 (10%)
Nosebleed	4 (6%)	0
≥ 4 days/week	1 (2%)	0
≥ 4 weeks consecutively	1 (2%)	0
Moderate-to-severe^a^	24 (37%)	2 (20%)
Mild^a^	15 (23%)	1 (10%)
Persistent^b^	3 (5%)	0
Intermittent^b^	36 (55%)	3 (30%)
Mean total symptom score	7.64 ± 3.79	11 ± 3.61
**Atopic dermatitis**	12 (18%)	0
**Single allergic disease**	31 (48%)	2 (20%)
**More than one allergic disease**	15 (23%)	1 (10%)

The differences between rDer p 23-positive and rDer p 23-negative groups were not statistically significant.

^a^Severity of allergic rhinitis: moderate-to-severe (disturbed sleep and/or impaired daily activities, sport, leisure, and/or impaired work and school and/or troublesome symptoms); mild (normal sleep, no impairment of daily activities, sports, leisure, no impairment of work and school and no troublesome symptoms).

^b^Persistence of allergic rhinitis: persistent (≥ 4 days per week and ≥ 4 weeks for any of the symptom of itchy nose, sneezing, runny nose, and nose blockage); intermittent (< 4 days per week or < 4 weeks for any of the symptom of itchy nose, sneezing, runny nose, and nose blockage).

*P* values were calculated using chi-square test or Fisher’s exact tests (for sample size < 5).

The IgE-binding residues analysis of rDer p 23 was carried out among the atopic individuals in the rDer p 23-positive group. The rDer p 23-positive group consisted of 46 atopic individuals with allergic diseases and 19 nonallergic control subjects. The 46 allergic individuals consisted of 48% (n = 31) with single allergic disease (AS/AR/AD only) and 23% (n = 15) with 2 or more allergic diseases (AS and AR, n = 5; AR and AD, n = 8; AS, AR and AD, n = 2). In the group, 18% (n = 12) fulfilled diagnostic criteria for AS, 60% (n = 39) for AR, and 18% (n = 12) for AD. The details of the allergic disease symptoms are shown in Table 1.

### Identification of IgE-binding residues of rDer p23

To identify IgE-binding residue of rDer p 23, the effect of each mutation on IgE binding was analyzed in comparison to the wild type rDer p 23 (set as 100%). The IgE binding capability of each mutant was tested for each of the 65 rDer p 23-sIgE positive sera. The percentage of IgE binding of each mutant relative to wild type rDer p 23 was calculated. A mutation was considered as significant if it resulted in more than 20% reduction in IgE binding as compared to the wild type rDer p 23. The median and range of percentage of IgE-binding reduction, and the percentage of sera which shows more than 20% IgE-binding reduction are shown (Supplementary Fig. S6). The immunoassay and the analysis of experimental data performed in this study have been previously used and validated in multiple other publications^12,15,16,25^. D56A mutant was however excluded from the analysis due to the high background in negative controls.

**Figure 3:**
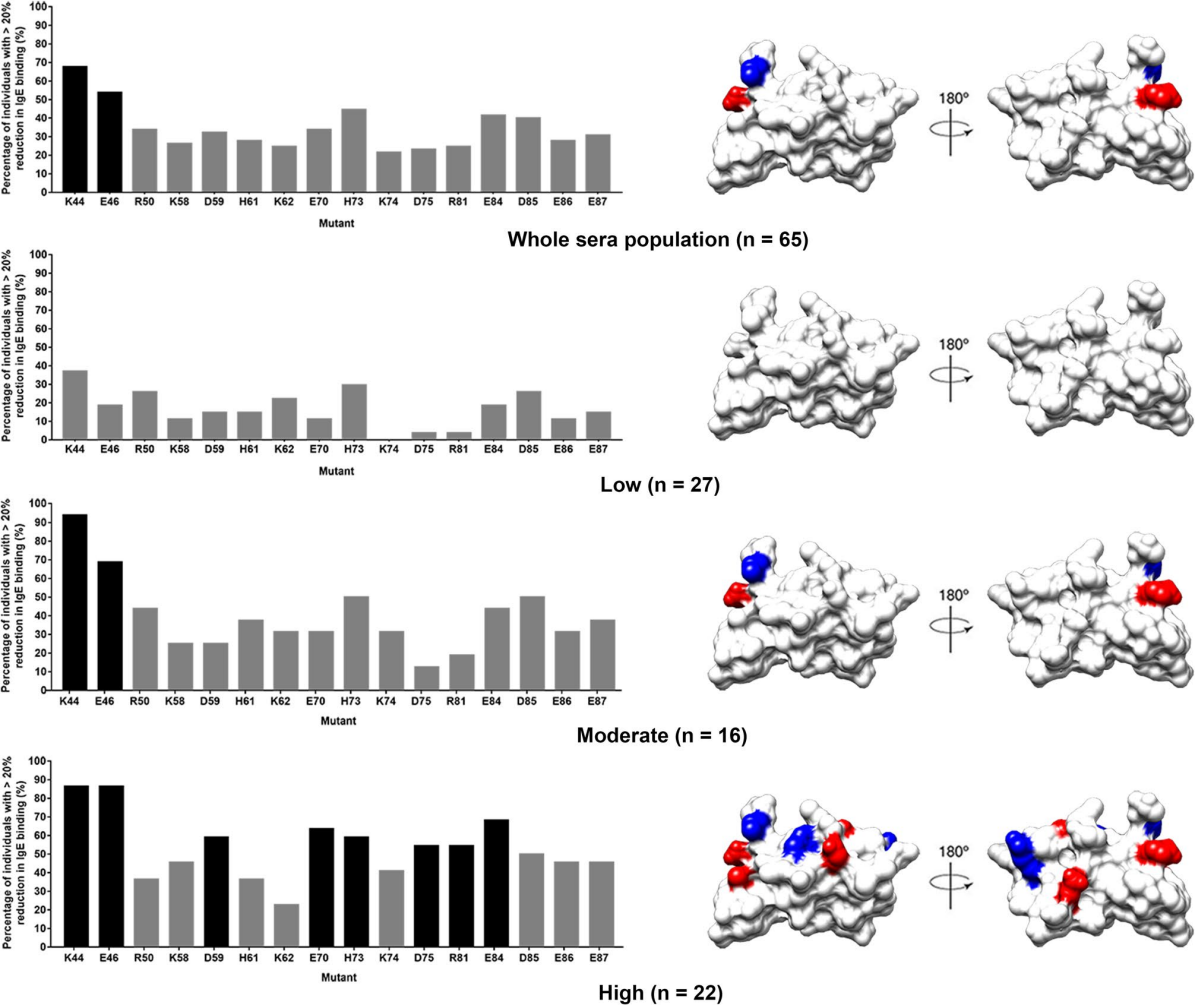
The site-directed mutagenesis for the determination of the IgE-binding residues of rDer p 23. Mutations that cause more than 20% reduction of the IgE binding in the individual sera are considered significant. Percentage of individuals showing > 20% reduction of IgE binding compared to wild type rDer p 23 was plotted for each mutant. Mutations that cause a significant reduction in the IgE binding in over 50% of individuals (the major IgE-binding residue) are highlighted in black and their location on the rDer p 23 structure is mapped (right panel). Graph (left panel) and surface diagram (right panel) showing the major IgE-binding residues for the whole population regardless of their rDerp23-sIgE levels, and the classified rDerp23-sIgE sera (low, moderate, and high IgE-binding intensity group). Blue: positively charged residues; Red: negatively charged residues. The electrostatic surface charge diagram was generated using program Chimera^37^.

The prevalence of these 16 mutations among 65 atopic individuals is shown in Fig. 3. Out of all the 16 residues mutated, only two mutations caused a significant IgE-binding reduction in more than half of the 65 sera (identified as the major IgE-binding residue), namely, K44 and E46. Mutation of K44 caused the most drastic reduction in IgE-binding, in which 68% (44 out of 65) sera showed more than 20% reduction in IgE-binding. Whereas, E46 mutant resulted in 54% (35 out of 65) sera IgE-binding reductions. The other 14 mutants showed IgE-binding reductions for less than 50% of the tested sera (range 22–45%). Furthermore, both K44 (26.1%) and E46 (21%) mutants showing higher median percentage of IgE-binding reduction compared to other mutations (Supplementary Fig. S6). Therefore, the results suggested that K44 and E46 are the major IgE-binding residues for rDer p 23 among the 65 atopic individuals.

### rDerp23-sIgE titers of atopic individuals correlate to number of IgE-binding residues

The 65 rDerp23-sIgE sera of different IgE-binding intensity were subsequently classified using the ImmunoCAP system into three groups, composed of low intensity level (range 23 < intensity < 232, equivalent to ImmunoCAP class 3; n = 27), moderate intensity level (range 232 < intensity < 460, equivalent to ImmunoCAP class 4 or 5; n = 16), and high intensity level (intensity > 460; equivalent to ImmunoCAP class 6; n = 22) (Table S4). The major IgE-binding residues in rDer p 23 were identified for each group.

**Figure 4:**
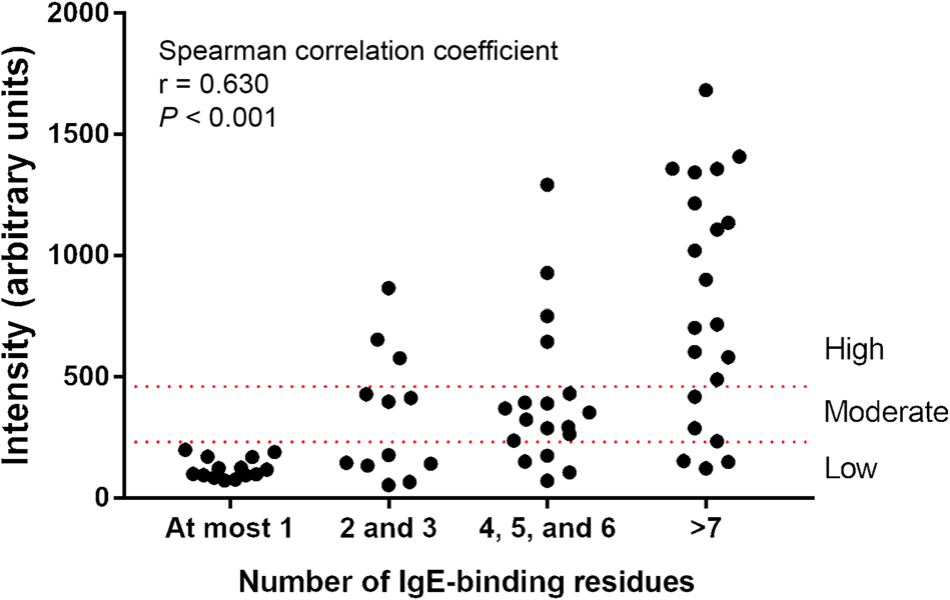
Nonparametric Spearman correlation analysis shows the positive correlation between intensity (arbitrary units) of rDer p 23-specific immunoglobulin E-positive individuals with the number of IgE-binding residues (Spearman correlation coefficient, r = 0.630; *P* < 0.001).

As shown in Fig. 3, the number of mutations contributing to a significant IgE-binding reduction in more than half of the 65 sera increases from low (n = 0) to moderate (n = 2), and then to high (n = 8) IgE-binding intensity group (highlighted in black). These major IgE-binding residues were mapped and found to be randomly distributed on the molecular surface of rDer p 23. This result suggests that the higher the rDer p 23-sIgE level in the sera, the higher the number of major IgE-binding residues identified for those respective groups. Moreover, the plot of IgE-binding intensity vs the number of IgE-binding residues showed that the rDer p 23-sIgE titers of atopic individuals are strongly correlated to the number of IgE-binding residues for rDer p 23 (Spearman correlation coefficient, r = 0.630; *P* < 0.001) (Fig. 4). Hence, the results show a clear positive correlation between the rDer p 23-sIgE titers and the number of IgE-binding residues for rDer p 23.

### Individuals who were only sensitized to HDM have a higher number of IgE-binding residues of rDer p 23 than individuals who were polysensitized to HDM and the other crude allergens

The 65 rDer p 23-positive sera were classified into two groups based on their SPT response to crude allergens (*D. pteronyssinus*, *B. tropicalis* (both are HDM), *Elaeis. guineensis* (tree pollen) and/or *Curvularia lunata* (fungus)). The two groups composed of a group that was only sensitized to HDM (*D. pteronyssinus* and/or *B. tropicalis*; n = 7) and a group that was polysensitized to HDM (*D. pteronyssinus* and/or *B. tropicalis*) and other crude allergens (*E. guineensis* and/or *C*. *lunata*) (n = 8). The major IgE-binding residues in rDer p 23 were identified for each group.

**Figure 5:**
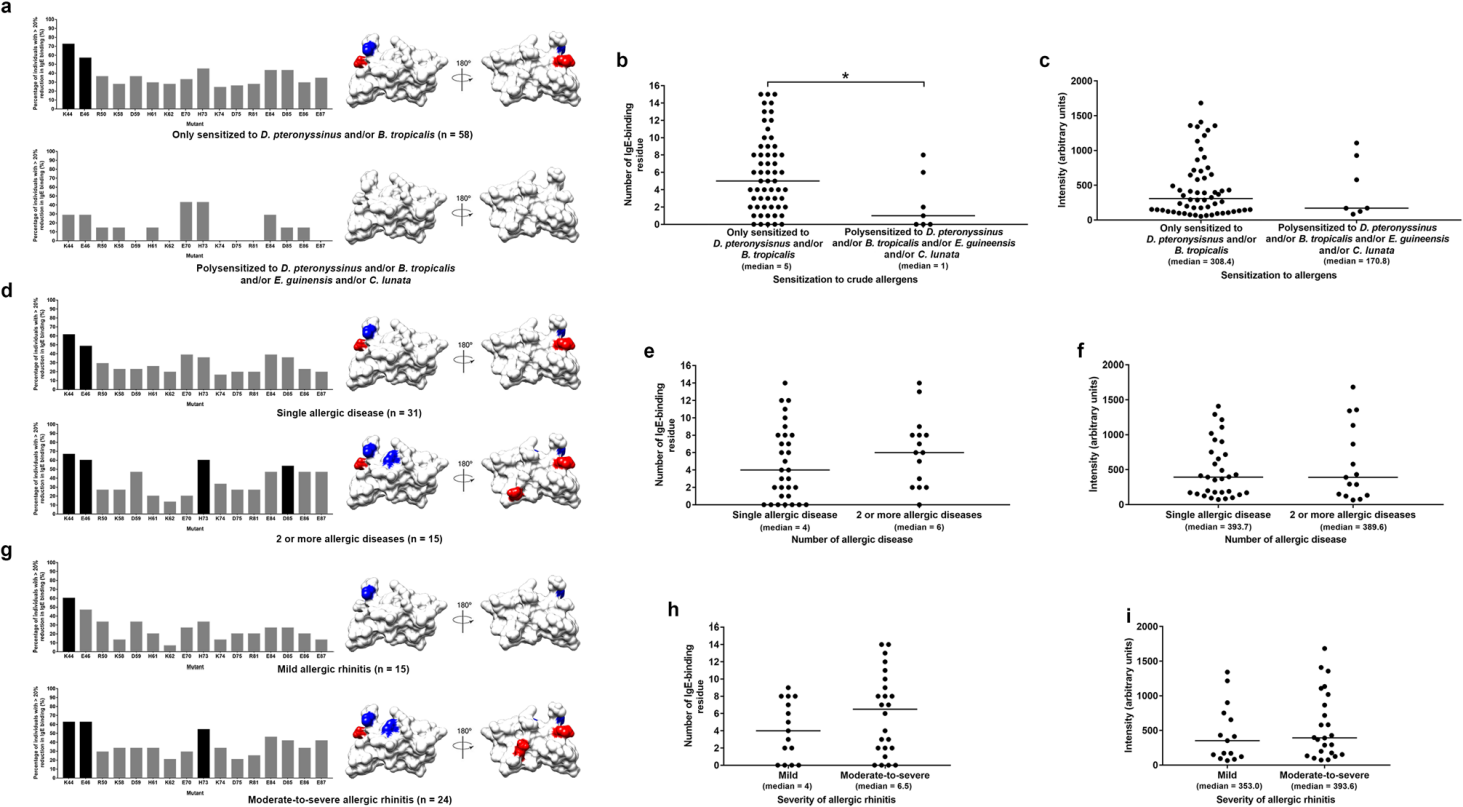
Analysis of major IgE-binding residues of rDer p 23 between allergic individuals with (**a**) different sensitization to crude allergen (sensitized to *D. pteronyssinus* and/or *B. tropicalis* vs polysensitized to *D. pteronyssinus* and/or *B. tropicalis* and/or *E. guineensis* and/or *C. lunata*) **(d**) allergic multimorbidity (single allergic disease vs 2 or more allergic diseases), and (**g**) severity of allergic rhinitis (mild vs moderate-to severe). Mutations that cause more than 20% reduction of the IgE binding in the individual sera are considered significant. Percentage of individuals showing > 20% reduction of IgE binding compared to wild type rDer p 23 was plotted for each mutant. Mutations that cause a significant reduction in the IgE binding in over 50% of individuals (the major IgE-binding residue) are highlighted in black (right panel) and their location on the rDer p 23 structure is mapped (left panel). Scatter plots showing (**b**, **e**, **h**) number of IgE-binding residues and (**c**, **f**, **i**) IgE-binding intensity for each comparison (median value are indicated). Blue: positively charged residues; Red: negatively charged residues. The electrostatic surface charge diagram was generated using program Chimera^37^.

As shown in Fig. 5a, the number of major IgE-binding residues for individuals in the group who were only sensitized to HDM (n = 2) is more than the group that was polysensitized to HDM and/or the other crude allergens (n = 0). This result suggests that the sIgE from individuals who were sensitized only to HDM recognize a higher number of major IgE-binding residue of rDer p 23. Moreover, the median value of the number of IgE-binding residues for the group that was only sensitized to HDM (median = 5) is significantly higher than the other group (median = 1) (*P* < 0.05) (Fig. 5b) although the IgE-binding intensities for both groups are similar (Fig. 5c). We also compared the number of IgE-binding residues and intensities between individuals who were monosensitized to *D. pteronyssinus* (n = 7) and cosensitized to both *D. pteronyssinus* and *B. tropicalis* (n = 51) (Supplementary Fig. S7). The number of IgE-binding residues and intensities for both groups are similar. Therefore, the results showed that the individuals who were monosensitized to *D. pteronyssinus* and cosensitized to HDM have a higher number of rDer p 23-sIgE than individuals who were polysensitized to HDM and the other crude allergens tested in this study.

### Allergic multimorbidity group has a higher number of IgE-binding residues than single allergic disease group

The 46 sera from individuals with allergic diseases composed of individuals with single allergic disease (AS only/AR only/AD only; n = 31) and allergic multimorbidity/coexistence of 2 or more allergic diseases (AS and AR, AS and AD, and AS, AR, and AD; n = 15) (Table 1). The major IgE-binding residues in rDer p 23 were determined for each group.

As shown in Fig. 5d, the number of major IgE-binding residues for individuals in 2 or more allergic diseases group (n = 4) is more than those in single allergic disease group (n = 2) (highlighted in black). These major IgE-binding residues were mapped on the molecular surface of rDer p 23. This result suggests that the sIgE from individuals with allergic multimorbidity recognize a higher number of major IgE-binding residues of rDer p 23. Moreover, the median value of the number of IgE-binding residues for the individuals in 2 or more allergic diseases group (median = 6) is higher than the other group (median = 4) (Fig. 5e) although the IgE-binding intensities for both the groups are similar (Fig. 5f). Taken together, the results showed that individuals with allergic multimorbidity have a higher number of rDer p 23-sIgE than individuals with single allergic disease.

### Moderate-to-severe AR group has a higher number of IgE-binding residues than mild AR group

We further determined the major IgE-binding residues among individuals with AR. The 39 individuals with AR were classified based on severity (mild; n = 15 vs moderate-to-severe; n = 24) according to ARIA guidelines^17^. Reported symptoms for each subject with AR are shown in Table 1. The major IgE-binding residues in rDer p 23 were determined for each group.

As shown in Fig. 5g, the number of major IgE-binding residues for individuals in moderate-to-severe AR group (n = 3) is more than mild AR group (n = 1) (highlighted in black). These major IgE-binding residues were mapped on the molecular surface of rDer p 23. Individuals in the moderate-to-severe AR group (median = 6.5) have a higher median value of the number of IgE-binding residues than mild AR group (median = 4) (Fig. 5h) although the IgE-binding intensities for moderate-to-severe and mild groups are similar (Fig. 5i). Thus, the results showed that individuals with moderate-to-severe AR type have a higher number of rDer p 23-sIgE than the mild AR type.

According to the ARIA guideline, AR can also be classified based on the duration of symptom. Of these 39 individuals with AR, there are three individuals with persistent AR as compared to 36 individuals with intermittent AR. The major IgE-binding residues in rDer p 23 were identified for each persistent and intermittent AR. The number of mutations contributing to a significant IgE-binding reduction in more than half of the sera with persistent AR (n = 7) is more than intermittent AR (n = 3) (Supplementary Fig. S7). Due to the small number of individuals with persistent AR type in this population, further study is needed to confirm this observation.

We also analyzed individuals with AR based on their combined symptom score for AR, using a scoring system that was modified from Adam et al.^28^ The mean total symptom score for individuals with AR in rDer p 23-positive group is 7.64 (Table 1). The 39 individuals with AR were classified into two groups based on the mean total symptom score, composed of the group of individuals that has total symptom score below the mean (n = 23) and above the mean (n = 16). As shown in Supplementary Fig. S7, both the groups have the same number of major IgE-binding residues (n = 2) and the median number of IgE-binding residues (median = 5). In addition, the median value of IgE-binding intensity for both the groups is also similar. Hence, there is no difference in the number of IgE-binding residues for individuals with a total symptom score for AR either below or above the mean.

### The duration of AR is correlated to the number of IgE-binding residues

To determine whether there is a correlation between the duration of an allergic disease and the number of IgE-binding residues, we have analyzed the number of IgE-binding residues for different durations of AR. As shown in Supplementary Fig. S8, there is an increase in the median value of the number of IgE-binding residues from 2.5 (1 to 4 years), 4.5 (5 to 10 years) to 7 (more than 10 years). The results showed that the longer the duration of AR disease, the higher the number of IgE-binding residues, although the correlation is not statistically significant. Therefore, larger sample sizes would be required in the future to confirm this observation. Additionally, we have analyzed the correlation between the duration of AR symptoms and the severity of AR disease. There is no association between the duration of AR symptoms and the severity of AR disease for both our sera population and the larger epidemiological cohort (data not shown).

## Discussion

In this study, we determined the prevalence rate of rDer p 23 and performed IgE-binding residues analysis on rDer p 23 using sera from atopic individuals. Our SPT results show a high sensitization rate of HDMs as compared to pollen and fungus allergen, indicating the importance of studying HDM allergens.

Surface-exposed polar or charged amino acid residues are the most abundant IgE-binding epitopes of proteins as shown in previous allergen epitope studies^10,12,15,16^. Based on the crystal structure of Der p 23, we have identified 17 surface-exposed and charged residues. Mutants that cause IgE-binding reduction for 65 atopic sera that have positive sIgE towards rDer p 23 allergen were determined. Our single-site mutagenesis and immuno-dot blot results indicated that two major IgE-binding residues (K44 and E46) were located at the N-terminal part of rDer p 23, a mutation of which was found to cause significant IgE-binding reduction for more than half of the studied population of 65 atopic sera (Fig. 3). Our finding is in contrast with the previous finding that the C-terminal part of Der p 23 allergen protein contains the major IgE epitopes^7^. This result suggests that different populations might have different major IgE-binding epitopes of rDer p 23. In this study population from a tropical region, residues K44 and E46 are the most important residues of rDer p 23 among those sera with moderate and high IgE-binding intensities (Fig. 3).

Mapping of IgE-binding residues on a rDer p 23 molecule showed that both the major IgE-binding residues K44 and E46 are located in proximity at the N-terminal region (~ 6.3 Å, the distance between Cα atoms). We postulated that both these residues are likely to involve in the formation of a major linear epitope of rDer p 23. This is due to the observation that the majority of the sera (56.1%; 32 out of 57) were found to have both these IgE-binding residues in common (Table S5). Besides, we also observe that both K44 and E46 could also be part of the other IgE epitope regions. For example, 42.1% (24 out of 57) sera were found to have both K44 and H73 as their IgE-binding residues. Based on the structure of rDer p 23, residues K44 and H74 (located at β3 strand) could be part of a conformational IgE-binding epitope (~ 10.6 Å, the distance between Cα atoms) (Table S5). Therefore, our single site mutagenesis data suggest that residues K44 and E46 not only could involve in forming the major linear epitope but also might involve in the formation of other linear and/or conformational epitopes of rDer p 23 (Table S5). Further studies with double mutations of both K44 and E46 residues may help to further determine the importance of these residues in IgE binding.

It is a well-established concept that allergic symptoms are triggered when the cross-linking of effector cells-bound IgE antibodies by allergens occurs. Thus, we further determine the possible epitope region of rDer p 23 that could facilitate the cross-linking of IgE. We observed that the IgE binding for a high percentage of (42.1%; 24 out of 57) sera seems to involve two pairs of distant residues E84 and K44/E46 (Supplementary Table S5). These pairs of residues are located at two opposite surfaces of rDer p 23 molecule, with E84 on one side and K44/E46 on the other side of the molecule (Fig. 1). We hypothesize that, in rDer p 23, residue E84 might form a cluster with amino acid residues on one side, whereas K44/E46 could form an epitope with other amino acids on the opposite side of the protein. This arrangement may allow binding of two IgE antibodies for cross-linking of the IgE receptor. Further studies with double/triple mutation are needed to identify the cluster of residues that involve in each of the epitope regions of rDer p 23.

We investigated the correlation between sera-sIgE titers and the number of IgE-binding residues of rDer p 23. Previously, in Der f 21 study, we have shown the direct correlation of the titer of sera-sIgE and the number of major epitope^15^. The results in this study provide further evidence that, the higher the allergen-sIgE level of an individual, the higher the number of major IgE-binding residues that are found in the allergen (Fig. 3). The allergen-sIgE titers are also strongly correlated to the number of IgE-binding residues for the allergen (Fig. 4). We also observed the random distribution of significant IgE-binding residues on the molecular structure of rDer p 23. Our studies further support the presence of a heterogeneous population of sIgE in the sera of atopic individuals in epitope recognition, as shown in Der f 21 study. The previous study on Der p 2 major allergen from group 2 mite allergen has also elucidated the positive correlation of Der p 2-sIgE sera titers and IgE-repertoire complexity^17^. Similarly, this study indicated the existence of the correlation between the sIgE titers of the atopic population and the number of IgE-binding residues in an aeroallergen.

In this study, atopic individuals were categorized to either monosensitized or cosensitized to HDM crude allergens. In addition, there are also atopic individuals who were polysensitized to HDM and the other tested crude allergens (tree pollen and fungus). To the best of our knowledge, there is a lack of IgE-binding residue analysis for aeroallergen among atopic individuals with different categories of sensitization. We reported that the individuals who were only sensitized to HDM have a significantly higher number of IgE-binding residues than individuals who were polysensitized to HDM and/or other crude allergens. The results suggested that the HDM-sensitized individuals who were only sensitized to HDM have a higher number sIgE antibodies towards HDM allergen component (rDer p 23 in this study), compared to individuals who were also sensitized to other crude allergens. Nonetheless, a larger sample size is required to validate the observation in this study.

The analysis of the number of major IgE-binding residues of rDer p 23 based on the complexity of allergic disease phenotype and severity of AR revealed that the number of IgE-binding residues of the rDer p 23 allergen is higher for individuals with 2 or more allergic diseases and moderate-to-severe AR. This demonstrates for the first time the clinical relevance of the number of IgE-binding residues for HDM allergy. For food allergies, the correlation between epitope diversity and severity and persistence of allergic reaction among patients with milk, egg, peanut, and wheat allergies has also been reported^29–35^.

Overall, our finding highlights the importance of applying the knowledge of the composition and complexity of allergen-sIgE repertoires to improve the prediction of the complexity of HDM allergic phenotype and severity of HDM allergic disease.

## Supplementary Information

Supplementary Information.

## Data Availability

The datasets generated or analyzed during this study are not publicly available due to individual privacy but are available from the corresponding author on reasonable request.

## Abbreviations

HDMs: House dust mites
sIgE: Specific immunoglobulin E
rDer p 23: Recombinant Der p 23
AS: Asthma
AR: Allergic rhinitis
AD: Atopic dermatitis
SPT: Skin prick test
ASA: Solvent-accessible surface area
GXG: Gly-X-Gly
Ni-NTA: Nickel-nitriloacetic acid
CD: Circular dichroism
