# Supplementary material for: IgE-binding residues analysis of the house dust mite allergen Der p 23
Source: Sci Rep. 2021 Jan 13;11:921. doi: 10.1038/s41598-020-79820-y (PMC7806929; doi:10.1038/s41598-020-79820-y)

**Supplementary information**

**IgE-binding residues analysis of the house dust mite allergen Der p 23**

**Sze Lei Pang^1‡,2^, Sri Anusha Matta^1^, Yang Yie Sio^1^, Yu Ting Ng^1^, Yee-How Say^3^, Chyan Leong Ng^2^* and Fook Tim Chew^1^***

^1^Department of Biological Sciences, National University of Singapore, 14 Science Drive 4, 117543, Singapore

^2^Institute of Systems Biology, Universiti Kebangsaan Malaysia, UKM Bangi, 43600 Selangor, Malaysia

^3^Department of Biomedical Science, Faculty of Science, Universiti Tunku Abdul Rahman (UTAR) Perak Campus, Kampar, Perak, 31900, Malaysia

*corresponding author email: [dbscft@nus.edu.sg](mailto:dbscft@nus.edu.sg) and [clng@ukm.edu.my](mailto:clng@ukm.edu.my)

**^‡^** Current address: Department of Biological Sciences, National University of Singapore, 14 Science Drive 4, 117543, Singapore

**Supplementary methods**

**Diagnostic criteria for asthma, allergic rhinitis, and atopic dermatitis**

AS was defined based on doctor-diagnosed asthma and a positive SPT response for HDM^1^. For AR, the diagnostic criteria were at least two of self-reported symptoms (itchy nose, sneezing, runny nose, and nose blockage) and a positive SPT response for HDM. AR was further classified, depending on severity (mild and moderate-to-severe) and duration of symptoms (intermittent and persistent) based on ARIA^2,3^. Severity of AR: moderate-to-severe (disturbed sleep and/or impaired daily activities, sport, leisure, and/or impaired work and school and/or troublesome symptoms); mild (normal sleep, no impairment of daily activities, sports, leisure, no impairment of work and school and no troublesome symptoms). Duration of symptoms: intermittent (< 4 days per week or < 4 weeks for any of the symptom of itchy nose, sneezing, runny nose, and nose blockage); persistent (≥ 4 days per week and ≥ 4 weeks for any of the symptom of itchy nose, sneezing, runny nose, and nose blockage). The symptom score for each individual with AR was analyzed using a scoring system modified from Adam et al.,^4^ that consisted of the combined scores of all 6 symptoms (itchy nose, sneezing, runny nose, nose blockage, snore, and nosebleed). The total possible combined score of 6 symptoms for an individual is from 0 (no symptom present) to 30 (maximum symptom score). Each symptom was scored using a 6-point rating scale (from 0 to 5), based on the presence of a symptom (absence/presence; 0 or 1), duration (intermittent/persistent; 0 or 1), and severity of the symptom scale (from 0 to 3). The score of a symptom (for example, itchy nose) for an individual is 5, if the symptom is present (1), persistent (1) and the severity of symptom scale (3). Atopic dermatitis cases were identified using the U.K Working Party’s Diagnostic Criteria for Atopic Dermatitis and ISAAC guidelines based on doctor-diagnosed symptoms of eczema^5,6^.

**Cloning, expression, and purification of rDer p 23**

The synthetic Der p 23 gene with codon-optimized for expression in *Escherichia coli* (*E. coli*) was produced and subcloned into pET-28b(+) expression vector between *Nco*I - *Xho*I restriction-endonuclease sites (GenScript, Piscataway, New Jersey, USA). The pET-28b(+)-Der p 23 construct was transformed into *E. coli* strain Rossetta-gami (DE3) cells to produce C-terminal 6 x His-tag fusion protein. Transformed bacteria cells were grown overnight in Luria-Bertani (LB) broth containing kanamycin (50 µg/mL) at 310 K. Overnight bacterial cultures were inoculated and grown in 1 L LB broth with kanamycin (50 µg/mL) and allowed to grow at 310 K until OD_600_ reached 0.6. The culture was induced by the addition of 0.5 mM isopropyl-β-D-thiogalactopyranoside and grown at 310 K before being harvested by centrifugation at 7459 x *g*. Pellet was resuspended in binding buffer containing 20 mM Tris-HCl (pH 7.9), 500 mM NaCl, 20 mM imidazole, and 20 mM β-mercaptoethanol. The cell suspension was lysed by sonication at an amplitude of 38% for 15 minutes (30 s pulse on and 30 s pulse off) followed by centrifugation at 17,200 x *g*. The supernatant containing Der p 23 protein was filter sterilized using a 0.22 µm PVDF membrane filters and then applied onto a nickel-nitrilotriacetic acid (Ni-NTA) coupled HisTrap HP 5 mL column, pre-equilibrated with binding buffer. The Der p 23 protein was eluted using a linear gradient of elution buffer (20 mM Tris-Cl, pH 7.9, 500 mM NaCl, 500 mM imidazole, and 20 mM β-mercaptoethanol). Eluted fractions containing Der p 23 protein were pooled and further purified by size-exclusion chromatography using a HiLoad 26/600 Superdex 75 pg gel filtration column (GE Healthcare) pre-equilibrated with size-exclusion buffer (20 mM Tris-HCl, pH 7.9, 500 mM NaCl, and 20 mM β-mercaptoethanol). The purified proteins fractions were then analyzed using 15% SDS-PAGE gel.

**Mass spectrometric analysis**

Peptide separation was performed using an Eksigent nanoLC Ultra and ChiPLC-nanoflex (Eksigent, Dublin, CA) in TrapElute configuration. Subsequently, the samples were loaded on a 0.5 mm x 200 μm column and eluted on an analytical 15 cm x 75 μm column (ChromXP C18-CL, 3 μm). A gradient formed by mobile phase A (2% (v/v) acetonitrile, 0.1% (v/v) formic acid) and mobile phase B (98% (v/v) acetonitrile, 0.1% (v/v) formic acid) was used to separate 2 μL of the sample. The flow rate to separate the sample was set at 0.3 μL/min. The following gradient elution was used for peptide separation: 0 to 5% of mobile phase B in 1 min, 5 to 12% of mobile phase B in 19 min, 12 to 30% of mobile phase B in 40 min, 30 to 90% of mobile phase B in 2 min, 90 to 90% in 7 min, 90 to 5% in 3 min and finally held at 5% of mobile phase B for 13 min. The tandem MS analysis was performed using a TripleTOF 5600 system (AB Sciex) under Information Dependent Mode. The mass range of 400-1800 m/z and accumulation times of 250 ms per spectrum were chosen for the precursor ions selection. MS/MS analysis was performed on the 20 most abundant precursors with an accumulation time of 100 ms per cycle. The dynamic exclusion time was at 15 s. Recording of MS/MS spectra was then acquired under high sensitivity mode with rolling collision energy.

**Protein identification**

Protein identification was carried out using ProteinPilot 5.0 software revision 4769 (AB Sciex), which uses the Paragon database search algorithm (5.0.0.0.4767) and the integrated false discovery rate (FDR) analysis function^7,8^. The obtained MS/MS spectra were searched using the user-defined search parameters: (a) Cysteine alkylation: with methyl methanethiosulfonate; (b) Digestion: Trypsin; (c) Instrument: TripleTOF 5600; (d) ID focus: Biological modifications; (e) Search effort: Thorough; (f) FDR analysis: Yes; (g) User modified parameter files: Yes. The data was searched against the protein sequence database (total 559,221 entries). A decoy database search strategy was applied to estimate the FDR for peptide identification. A cutoff of unused protein score > 1.3 (95% confidence) was applied for protein identification.

**Generation of rDer p 23 mutants**

The program AREAIMOL of the CCP4 suites^9^ was used to calculate the solvent-accessible surface area (ASA) of Der p 23 dimer protein (PDB code: 4ZCE)^10^. The obtained ASAs were normalized to the corresponding Gly-X-Gly (GXG) tripeptide for a more accurate calculation^11^. The ratio of ASA to calculated GXG value of polar charged amino acid residues for Der p 23 was obtained for chain A and B. A total of 17 mutants with alanine substitution were synthesized (GenScript, Piscataway, New Jersey, USA) and subcloned in pET 28b(+) expression vector. These 17 constructs were transformed into *E. coli* strain Rosetta-gami (DE3) cells to produce C-terminal 6 x His-tag fusion protein. Transformed bacterial cells were grown overnight at 310K in LB broth containing kanamycin (50 μg/mL). His-tagged rDer p 23 mutants were then expressed and purified using Ni-NTA affinity chromatography as described above.

**Circular dichorism**

Circular dichorism (CD) experiments were conducted with 7 µM of protein in water at room temperature. Far-UV CD spectra were acquired with Jasco J-1500 CD spectrometer using a quartz cuvette with a 0.1 cm path length. The spectra were recorded at a wavelength range of 190-260 nm with 0.1 nm resolution using a scan speed of 100 nm/min. Each spectrum was normalized to mdeg = 0 at wavelength 260 nm. Data were converted to mean residue weight ellipticity (deg cm^2^ dmol^-1^). The CD data were further smoothened and analyzed with CAPITO tool^12^.

**Supplementary figures**


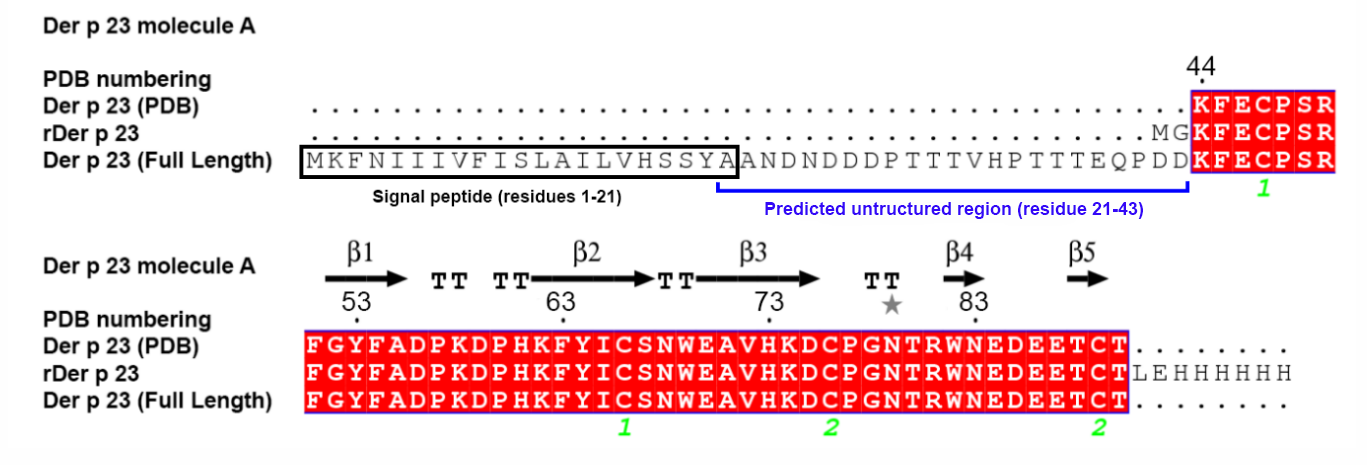


**Supplementary Fig S1.** Comparison among Der p 23 protein sequences. The sequence of Der p 23 from PDB file (Der p 23 (PDB)) (PDB code: 4ZCE)^8^ was aligned with rDer p 23 (name of construct in this study) and complete amino acid of Der p 23 (Der p 23 (Full length)) (Accession number: ACB46292)^13^. The reported signal peptide sequences and unstructured region of Der p 23 (full length) were indicated below the sequences. The conserved amino acid residues are colored in white with a red background. The secondary structures of Der p 23 sequence, according to the structure of Der p 23 (PDB code: 4ZCE) are highlighted above the alignment. Five β sheets β1, β2, β3, β4, and β5 are rendered as arrows, whereas strict β-turns as TT letters. Grey star on top of N79 residue indicates that N79 has alternate conformations. The numbering of Der p 23 (PDB) according to the PDB file is shown on the top. The green letter (***1***, ***1***, ***2*** and ***2***) under the cysteine residues that form disulphide bridges. The sequence alignment was performed using ClustalW^14^ and the figure was prepared using ESPript 3.0^15^.


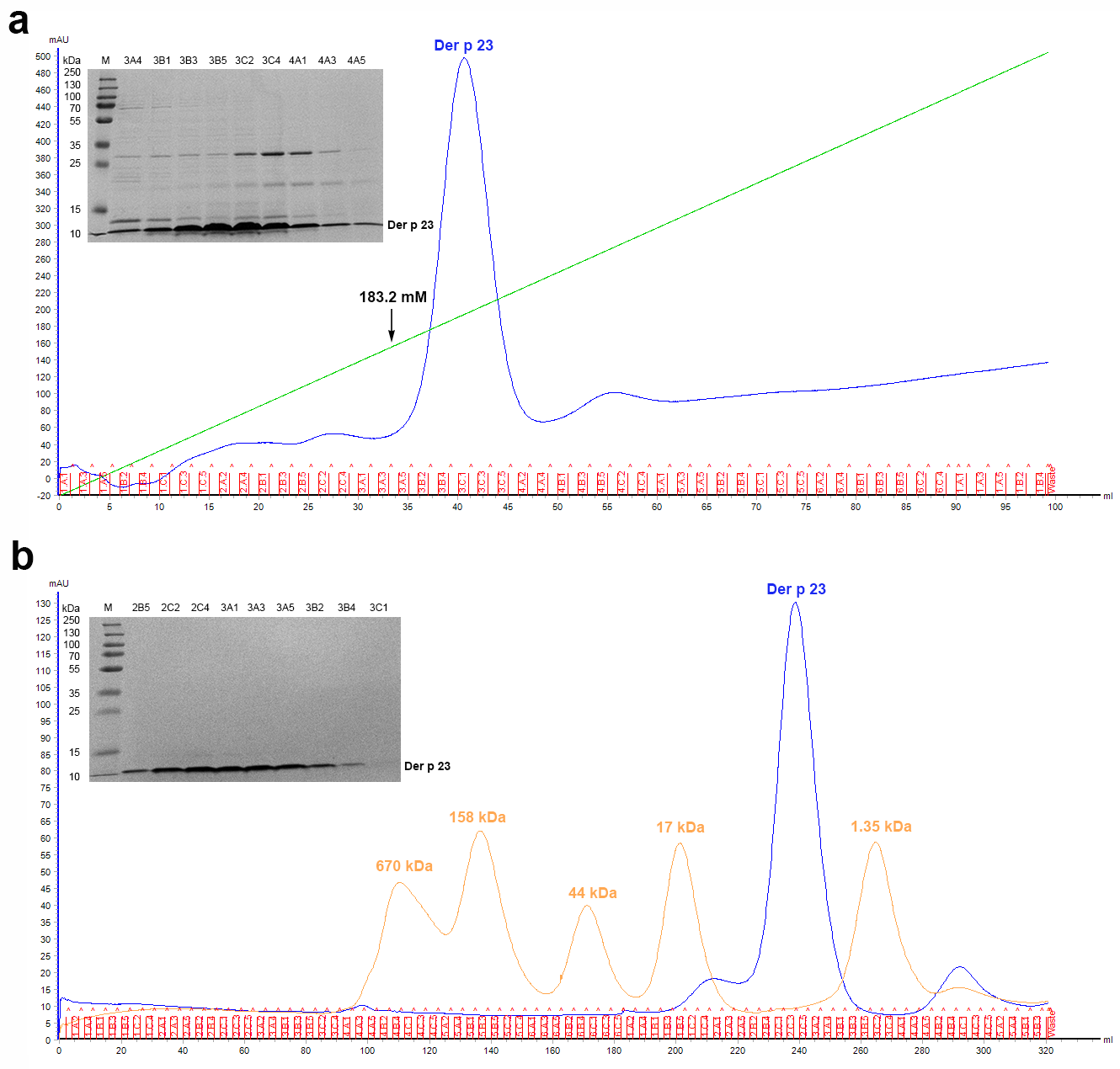


**Supplementary Fig S2.** Affinity chromatography, size exclusion chromatography (SEC) and 15% SDS-PAGE profiles of rDer p 23. **a** Affinity chromatography and 15% SDS-PAGE profile shows that rDer p 23 was eluted at 183.2 mM imidazole. **b** SEC and 15% SDS-PAGE profile shows that rDer p 23 was eluted at retention volume of 238.6 mL that corresponded to the molecular weight of 3.57 kDa. M: PageRuler^TM^ Plus Prestained protein ladder (Thermo Scientific).


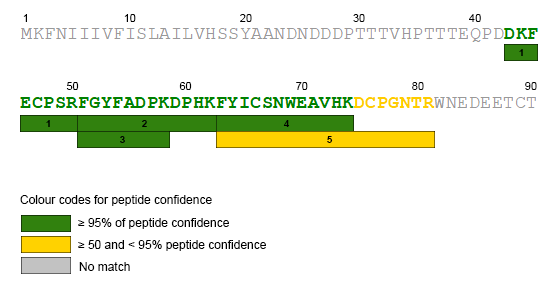


**Supplementary Fig S3.** Matched peptide sequences of rDer p 23 obtained using the TripleTOF 5600 (AB Sciex) mass spectrometer. Protein sequence coverage (% Cov (95)) is 35.6%. The identified sections of sequences are color-coded. Peptides identified with high confidence (>95%) are green, while peptides with moderate confidence (between 50-95%) are yellow. Peptides in grey indicate portions of sequences with no spectral evidence.


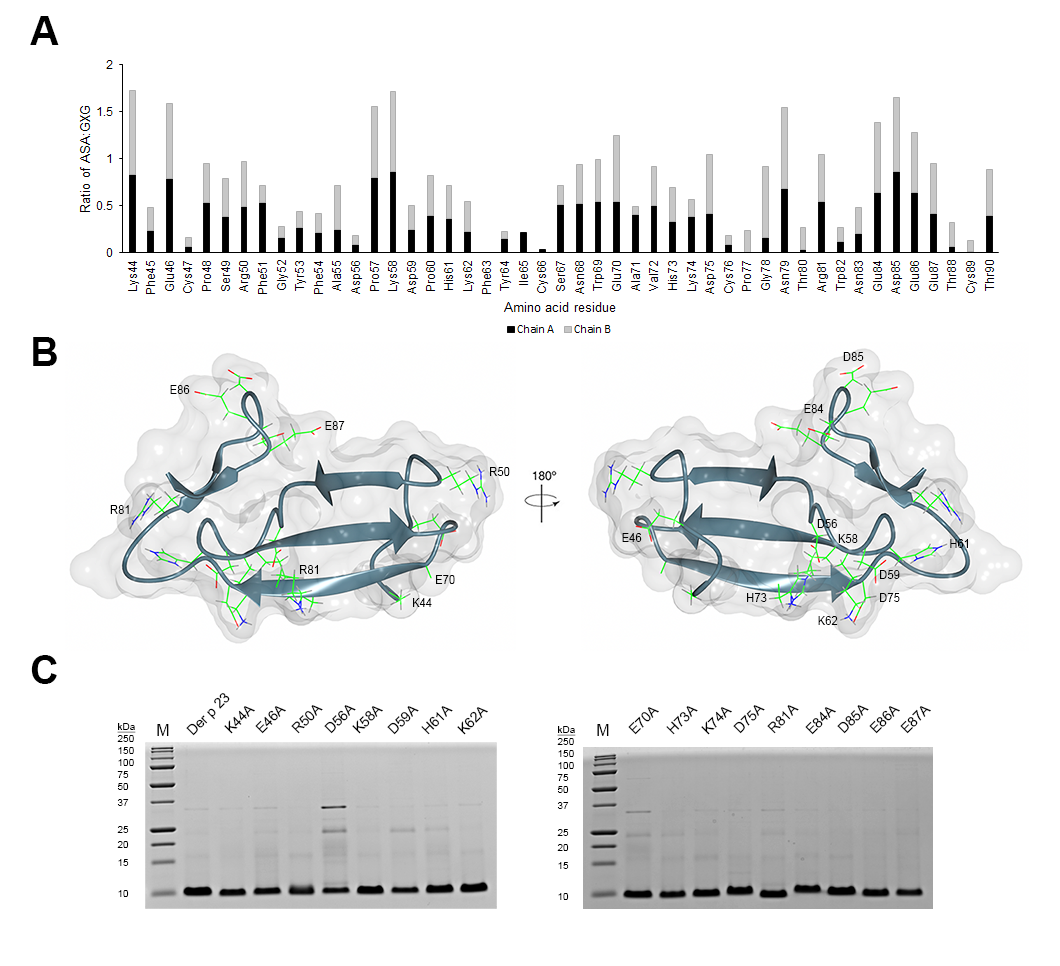


**Supplementary Fig S4.** Tricine-sodium dodecyl sulfate-polyacrylamide gel electrophoresis (SDS-PAGE) of the wild type rDer p 23 and 17 solvent-accessible, polar, and charged amino acid residues selected for the mutagenesis. 15% tricine-SDS-PAGE gels showing all 17 mutant proteins after being purified using affinity chromatography. M: PageRuler^TM^ Plus Prestained protein ladder (Thermo Scientific).

**
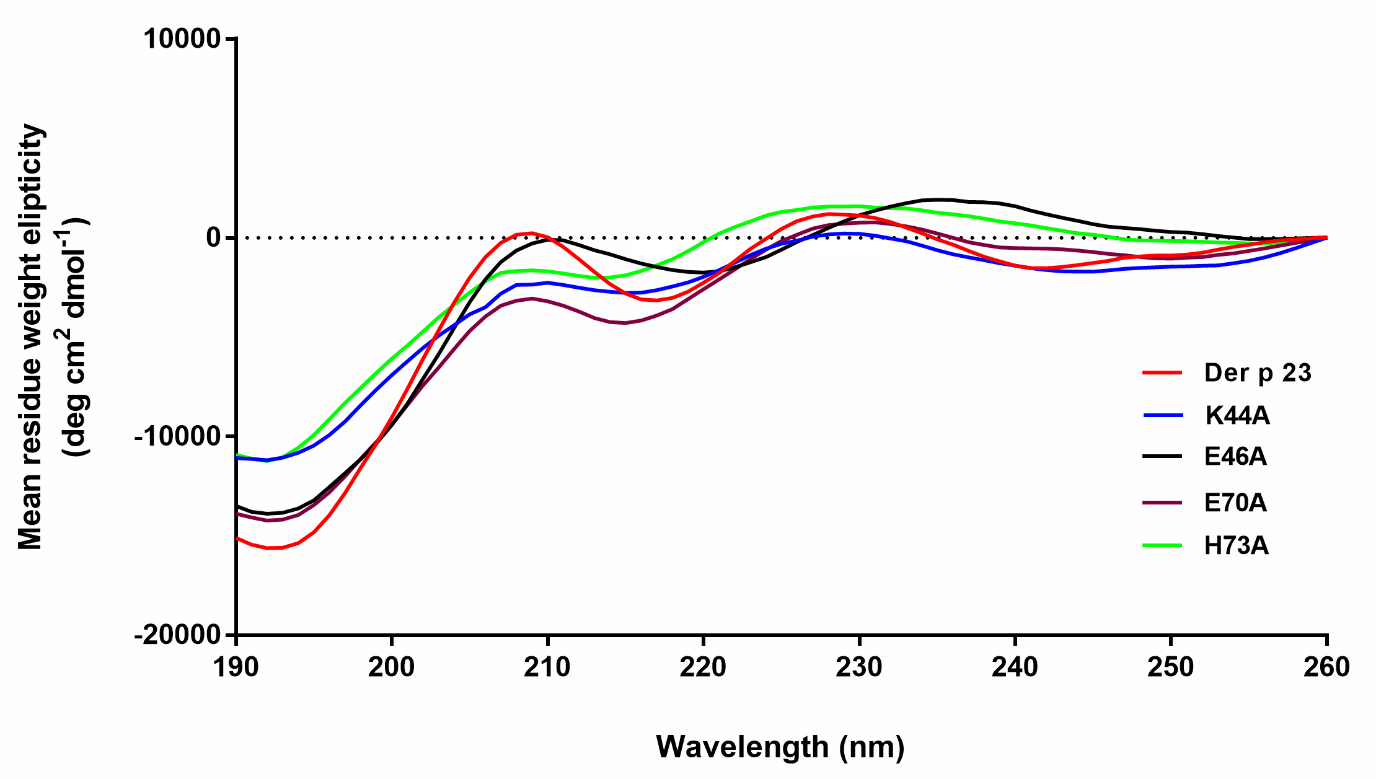
**

**Supplementary Fig S5.** Circular dichorism spectra of wild type Der p 23 and Der p 23 mutants. The far-UV CD spectra of the mutants of Der p 23 closely resemble of the wild type Der p 23 protein, with their predominant random coil secondary structure bearing the minima around 195 nm. The CD spectra were smoothed using the CAPITO tool^12^.

**
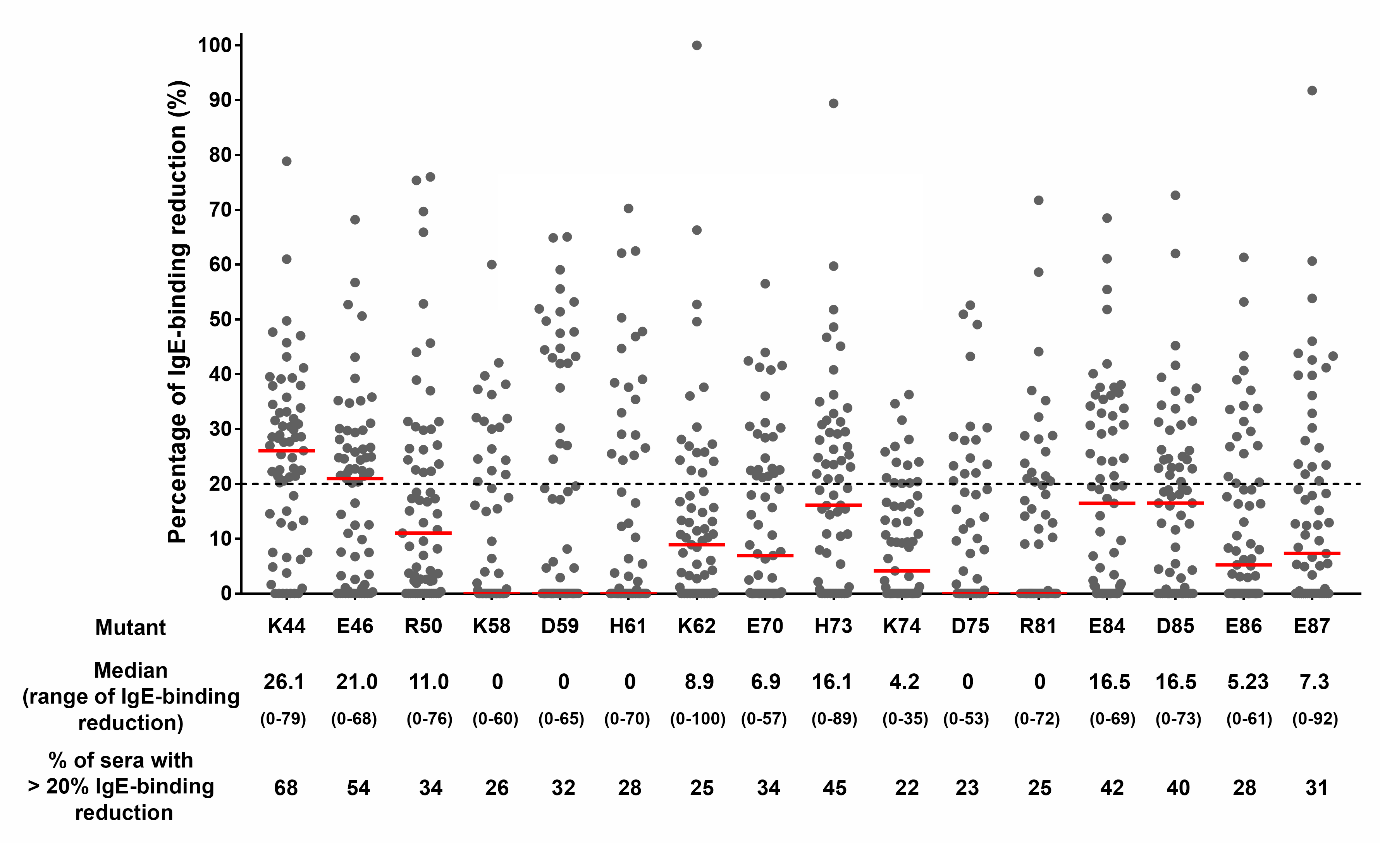
**

**Supplementary Fig S6.** Percentage of IgE-binding reduction of 65 sera for each of the rDer p 23 mutant (median values were indicated with red line). The median value including range of IgE-binding reduction is shown. The percentage of sera with > 20% of IgE-binding reduction is calculated.

**
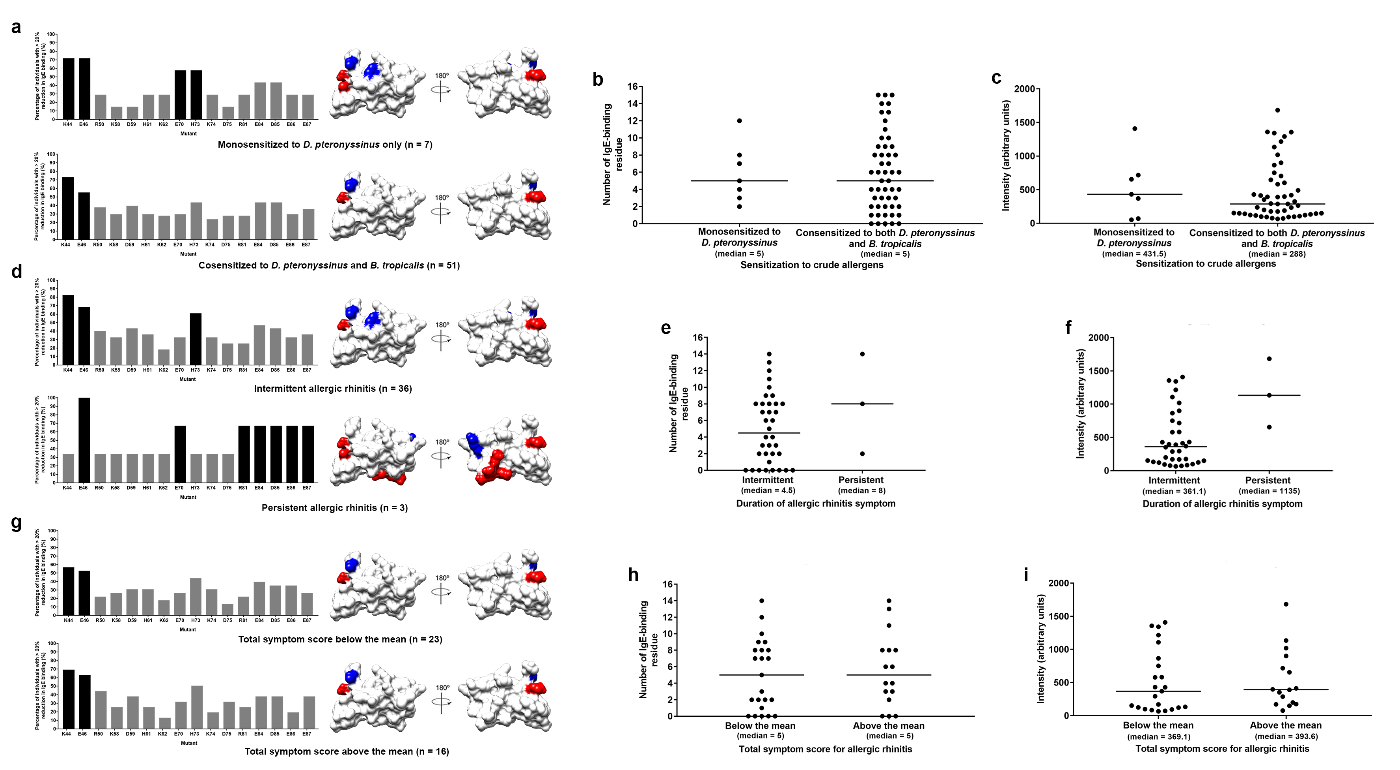
**

**Supplementary Fig S7.** Analysis of major IgE-binding residues of rDer p 23 between allergic individuals with **a** different sensitization to the crude allergen (monosensitized to *D. pteronyssinus* only vs cosensitized to both *D. pteronyssinus* and *B. tropicalis*) **d** different duration of allergic rhinitis (intermittent vs persistent) and **g** different total symptom score for allergic rhinitis (total symptom score below the mean vs above the mean). Mutations that cause more than 20% reduction of the IgE binding in the individual sera are considered significant. Percentage of individuals showing >20% reduction of IgE binding compared to wild type rDer p 23 was plotted for each mutant. Mutations that cause a significant reduction in the IgE binding in over 50% of individuals (the major IgE-binding residue) are highlighted in black (right panel) and their location on the rDer p 23 structure is mapped (left panel). Scatter plots showing **b, e, h** number of IgE-binding residues and **c, f, i** IgE-binding intensity for each comparison (median value are indicated). Blue: positively charged residues; Red: negatively charged residues. The electrostatic surface charge diagram was generated using program Chimera^16^.

**
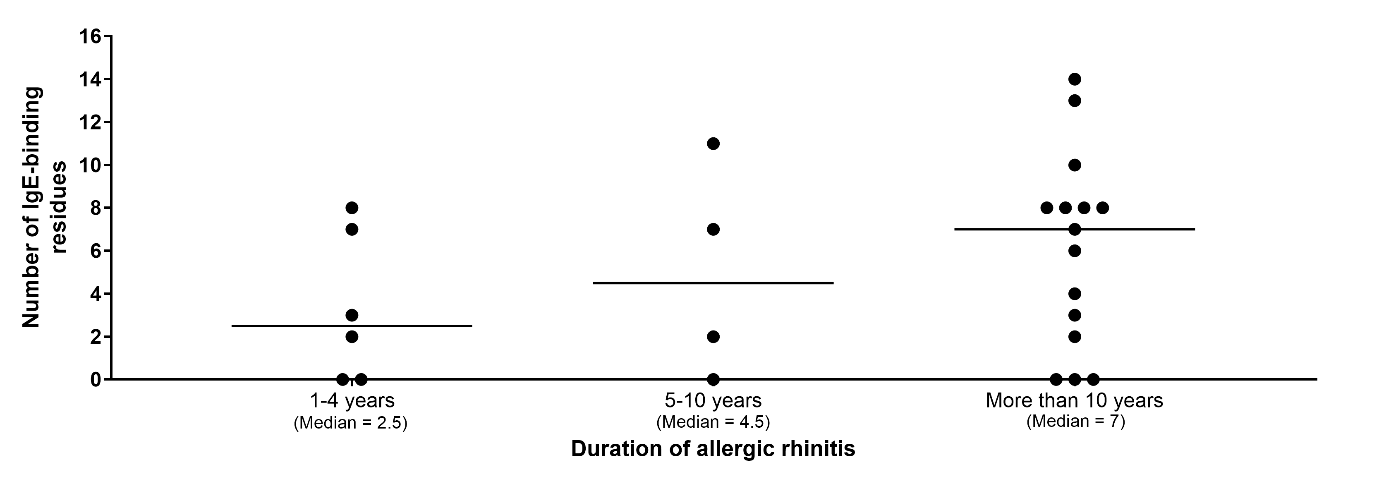
**

**Supplementary Fig S8.** Comparison of the number of IgE-binding residues for different duration of allergic rhinitis. There is an increase in the median number of IgE-binding residues from 1 to 4 years to more than 10 years of allergic rhinitis disease.


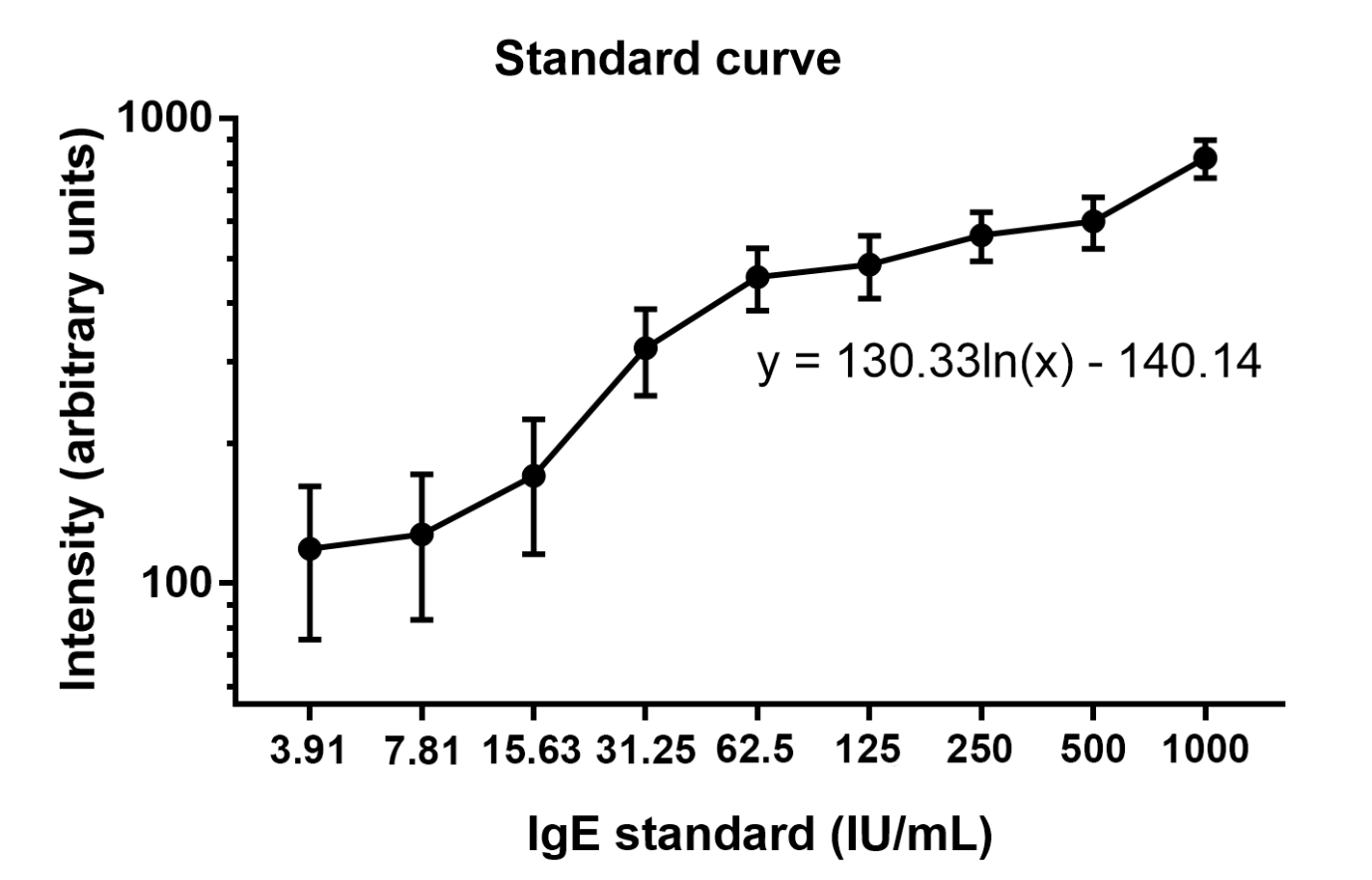


**Supplementary Fig S9.** IgE standard curve for the determination of Der p 23-specific IgE in human serum was obtained by using immuno-dot blot assay. The mean (95% CI) of the intensity (arbitrary units) was plotted against the IgE standard (IU/mL). The logarithmic equation is needed for the determination of Derp23-specific IgE in human serum.

**Supplementary tables**

**Table S1** Identified proteins based on tryptic digested peptides of rDer p 23 using the TripleTOF 5600 mass spectrometry

| ^a^N | ^b^Unused ProtScore | ^c^Total ProtScore | ^d^% Cov | ^e^% Cov (50) | ^f^% Cov (95) | ^g^Accession | ^h^Name | ^i^Species | ^j^Peptides (95%) |
| --- | --- | --- | --- | --- | --- | --- | --- | --- | --- |
| 1 | 13.98 | 13.98 | 43.33 | 43.33 | 35.56 | sp\|L7N6F8 | Major mite allergen Der p 23 | *Dermatophagoides pteronyssinus* | 56 |
| 2 | 8 | 8 | 41.75 | 41.75 | 41.75 | sp\|Q3YWT8 | 30S ribosomal protein S10 | *Shigella sonnei* | 4 |
| 3 | 7.67 | 7.67 | 44.59 | 40.26 | 40.26 | sp\|P00761 | Trypsin | *Sus scrofa* | 21 |
| 4 | 6 | 6 | 28.71 | 28.71 | 28.71 | sp\|Q3YWV2 | 30S ribosomal protein S14 | *Shigella sonnei* | 4 |

^a^N: the rank of the specified protein relative to all other proteins in the detected list.

^b^Unused Protscore/ Unused protein score: a measure of the protein confidence for a detected protein, calculated from the peptide confidence for peptides from spectra that are not already completely “used” by higher scoring winning proteins.

^c^Total ProtScore/ Total protein score: a measure of the total amount of evidence for a detected protein. The Total ProtScore is calculated using all of the peptides detected for the protein. The Total ProtScore does not indicate anything about the confidence that a protein has been detected, because some or even all of the spectra contributing to the Total ProtScore may be better explained by higher ranked proteins.
^d^% Cov (coverage): The percentage of matching amino acids from identified peptides having confidence greater than 0% divided by the total number of amino acids in the sequence.

^e^% Cov (50): The percentage of matching amino acids from identified peptides having confidence greater than or equal to 50% divided by the total number of amino acids in the sequence.

^f^% Cov (95): The percentage of matching amino acids from identified peptides having confidence greater than or equal to 95% divided by the total number of amino acids in the sequence.

^g^Accession: The accession number for the protein.

^h^Name: The name of this protein.

^i^Species: The species of this protein.

^j^Peptides (95%): The number of distinct peptides that have at least 95% confidence. Multiple modified and cleaved states of the same underlying peptide sequence are considered as distinct peptides because they have different molecular formulas. Multiple spectra of the same peptide that due to replicate acquisition or different charge states, only count once.

**Table S2** Tryptic fragments and the corresponding mass value for rDer p 23 peptide sequences

| **Tryptic fragment** | **Residue** | **Sequence** | **Theoretical mass** | **Experimental mass** |
| --- | --- | --- | --- | --- |
| 1 | 43-50 | DKFECPSR | 514.22 | 514.22 |
| 2 | 51-62 | FGYFADPKDPHK | 472.57 | 472.57 |
| 3 | 51-58 | FGYFADPK | 474.73 | 474.73 |
| 4 | 63-74 | FYICSNWEAVHK | 514.90 | 514.90 |
| 5 | 63-81 | FYICSNWEAVHKDCPGNTR | 590.01 | 590.02 |

**Table S3** Table shows the percentages of secondary structures of wild type Der p 23 and mutants following CAPITO analysis of spectra

| **Protein name** | **Protein secondary structure prediction (%)** | | |
| --- | --- | --- | --- |
|  | **Helix** | **β-strand** | **Random coil** |
| Der p 23 | 5 | 51 | 66 |
| K44A | 5 | 44 | 61 |
| E46A | 6 | 45 | 66 |
| E70A | 4 | 45 | 66 |
| H73A | 6.6 | 44 | 63 |

**Table S4** Intensity (arbitrary unit) derived using the logarithmic equation and the corresponding ImmunoCAP class

| **ImmunoCAP** | | **Intensity (a.u.)** | **Level of intensity** |
| --- | --- | --- | --- |
| **Class** | **IU/mL** |  |  |
| 3 | 3.5 to <17.5 | 23 to <232 | Low |
| 4 | 17.5 to <50 | 232 to <369 | Moderate |
| 5 | 50 to <100 | 369 to <460 | Moderate |
| 6 | >100 | >460 | High |

**Table S5** Matrix table showing the percentages of individuals who have the combination of IgE-binding residues (x- and y- axis). The increase in the percentages of combination is marked with colors from red to white for each of the cells.

|  | **K44** | **E46** | **R50** | **K58** | **D59** | **H61** | **K62** | **E70** | **H73** | **K74** | **D75** | **R81** | **E84** | **D85** | **E86** | **E87** |
| --- | --- | --- | --- | --- | --- | --- | --- | --- | --- | --- | --- | --- | --- | --- | --- | --- |
| **K44** |  |  |  |  |  |  |  |  |  |  |  |  |  |  |  |  |
| **E46** | 56.1 |  |  |  |  |  |  |  |  |  |  |  |  |  |  |  |
| **R50** | 35.1 | 24.6 |  |  |  |  |  |  |  |  |  |  |  |  |  |  |
| **K58** | 29.8 | 28.1 | 17.5 |  |  |  |  |  |  |  |  |  |  |  |  |  |
| **D59** | 35.1 | 31.6 | 24.6 | 24.6 |  |  |  |  |  |  |  |  |  |  |  |  |
| **H61** | 24.6 | 26.3 | 14.0 | 19.3 | 17.5 |  |  |  |  |  |  |  |  |  |  |  |
| **K62** | 19.3 | 17.5 | 5.3 | 8.8 | 7.0 | 10.5 |  |  |  |  |  |  |  |  |  |  |
| **E70** | 31.6 | 28.1 | 19.3 | 14.0 | 17.5 | 10.5 | 12.3 |  |  |  |  |  |  |  |  |  |
| **H73** | 42.1 | 29.8 | 31.6 | 17.5 | 28.1 | 15.8 | 10.5 | 24.6 |  |  |  |  |  |  |  |  |
| **K74** | 24.6 | 22.8 | 12.3 | 17.5 | 17.5 | 14.0 | 5.3 | 12.3 | 17.5 |  |  |  |  |  |  |  |
| **D75** | 26.3 | 22.8 | 15.8 | 14.0 | 22.8 | 10.5 | 5.3 | 19.3 | 21.1 | 14.0 |  |  |  |  |  |  |
| **R81** | 26.3 | 26.3 | 12.3 | 17.5 | 19.3 | 17.5 | 10.5 | 15.8 | 19.3 | 17.5 | 17.5 |  |  |  |  |  |
| **E84** | 42.1 | 42.1 | 19.3 | 22.8 | 24.6 | 28.1 | 14.0 | 17.5 | 22.8 | 17.5 | 19.3 | 22.8 |  |  |  |  |
| **D85** | 38.6 | 33.3 | 21.1 | 28.1 | 26.3 | 22.8 | 14.0 | 17.5 | 22.8 | 17.5 | 15.8 | 19.3 | 33.3 |  |  |  |
| **E86** | 28.1 | 24.6 | 15.8 | 17.5 | 21.1 | 17.5 | 8.8 | 19.3 | 15.8 | 15.8 | 17.5 | 15.8 | 26.3 | 26.3 |  |  |
| **E87** | 31.6 | 29.8 | 24.6 | 22.8 | 29.8 | 19.3 | 10.5 | 15.8 | 24.6 | 17.5 | 17.5 | 17.5 | 26.3 | 28.1 | 22.8 |  |

**References**

1. Andiappan, A.K. *et al.* Functional variants of 17q12-21 are associated with allergic asthma but not allergic rhinitis. *J Allergy Clin Immunol.* **137**, 758-766. e753 (2016).

2. Brożek, J.L. *et al.* Allergic Rhinitis and its Impact on Asthma (ARIA) guidelines: 2010 revision. *J Allergy Clin Immunol.* **126**, 466-476 (2010).

3. Bousquet, J. *et al.* World health organization; GA (2) LEN; AllerGen. allergic rhinitis and its impact on asthma (ARIA) 2008 update (in collaboration with the world health organization, GA (2) LEN and AllerGen). (2008).

4. Adam, P., Stiffman, M. & Blake Jr, R.L. A clinical trial of hypertonic saline nasal spray in subjects with the common cold or rhinosinusitis. *Arch Fam Med.* **7**, 39 (1998).

5. Asher, M. *et al.* International Study of Asthma and Allergies in Childhood (ISAAC): rationale and methods. *Eur Respir J.* **8**, 483-491 (1995).

6. Williams, H., Jburney, P., Pembroke, A., Hay, R. & Party, A.D.D.C.W. The UK Working Party's diagnostic criteria for atopic dermatitis. III. Independent hospital validation. *Br J Dermatol.* **131**, 406-416 (1994).

7. Shilov, I.V. *et al.* The Paragon Algorithm, a next generation search engine that uses sequence temperature values and feature probabilities to identify peptides from tandem mass spectra. *Mol Cell Proteomics* **6**, 1638-1655 (2007).

8. Tang, W.H., Shilov, I.V. & Seymour, S.L. Nonlinear fitting method for determining local false discovery rates from decoy database searches. *J Proteome Res.* **7**, 3661-3667 (2008).

9. Winn, M.D. *et al.* Overview of the CCP4 suite and current developments. *Acta Crystallogr D Biol Crystallogr.* **67**, 235-242 (2011).

10. Mueller, G.A. *et al.* Serological, genomic and structural analyses of the major mite allergen Der p 23. *Clin Exp Allergy* **46**, 365-376 (2016).

11. Miller, S., Janin, J., Lesk, A.M. & Chothia, C. Interior and surface of monomeric proteins. *J Mol Biol.* **196**, 641-656 (1987).

12. Wiedemann, C., Bellstedt, P. & Görlach, M. CAPITO—a web server-based analysis and plotting tool for circular dichroism data. *Bioinformatics* **29**, 1750-1757 (2013).

13. Weghofer, M. *et al.* Identification of Der p 23, a peritrophin-like protein, as a new major Dermatophagoides pteronyssinus allergen associated with the peritrophic matrix of mite fecal pellets. *J. Immunol*. **190**, 3059-3067 (2013).

14. Thompson, J.D., Gibson, T.J. & Higgins, D.G. Multiple sequence alignment using ClustalW and ClustalX. *Curr Protoc Bioinformatics*. **1**, 2-3 (2003).

15. Robert, X. & Gouet, P. Deciphering key features in protein structures with the new ENDscript server. *Nucleic Acids Res.* **42**, W320-W324 (2014).

16. Sanner, M.F., Olson, A.J. & Spehner, J.C. Reduced surface: an efficient way to compute molecular surfaces. *Biopolymers.* **38**, 305-320 (1996).

**Full SDS-PAGE images for Supplementary Fig S2**


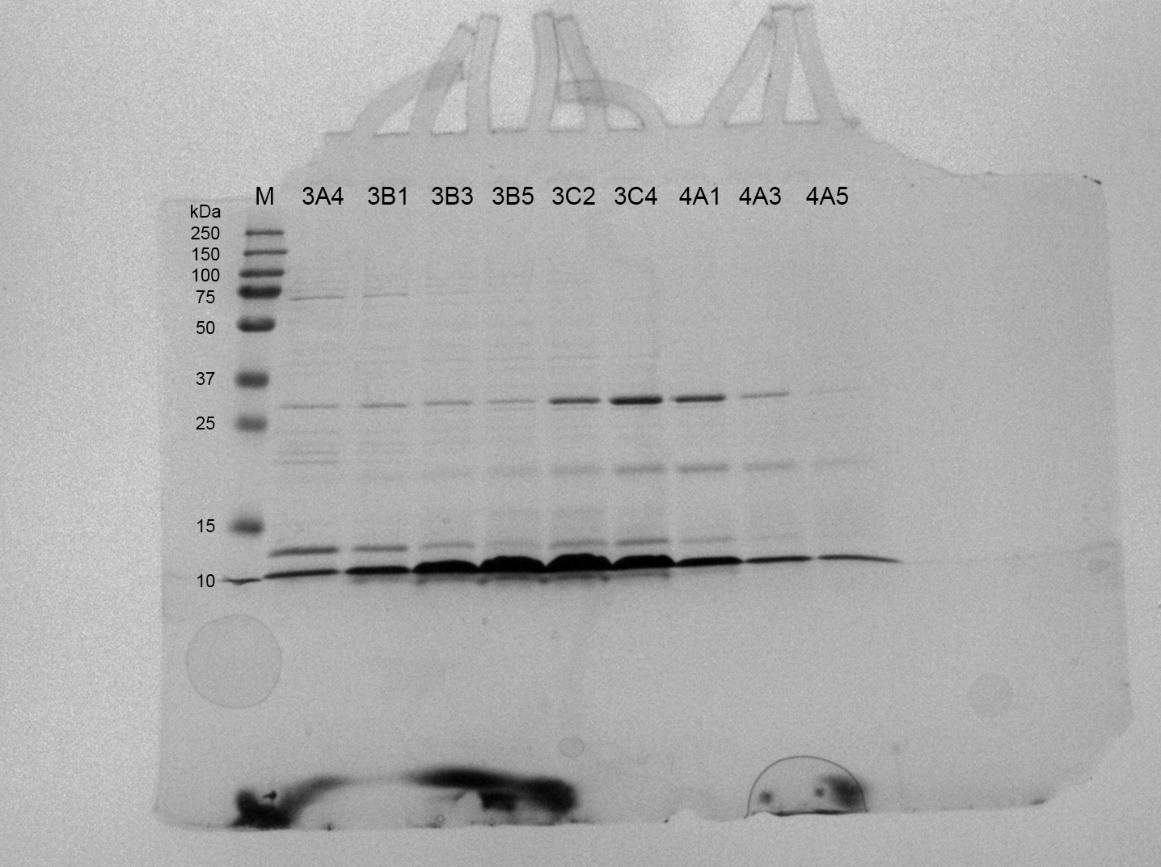


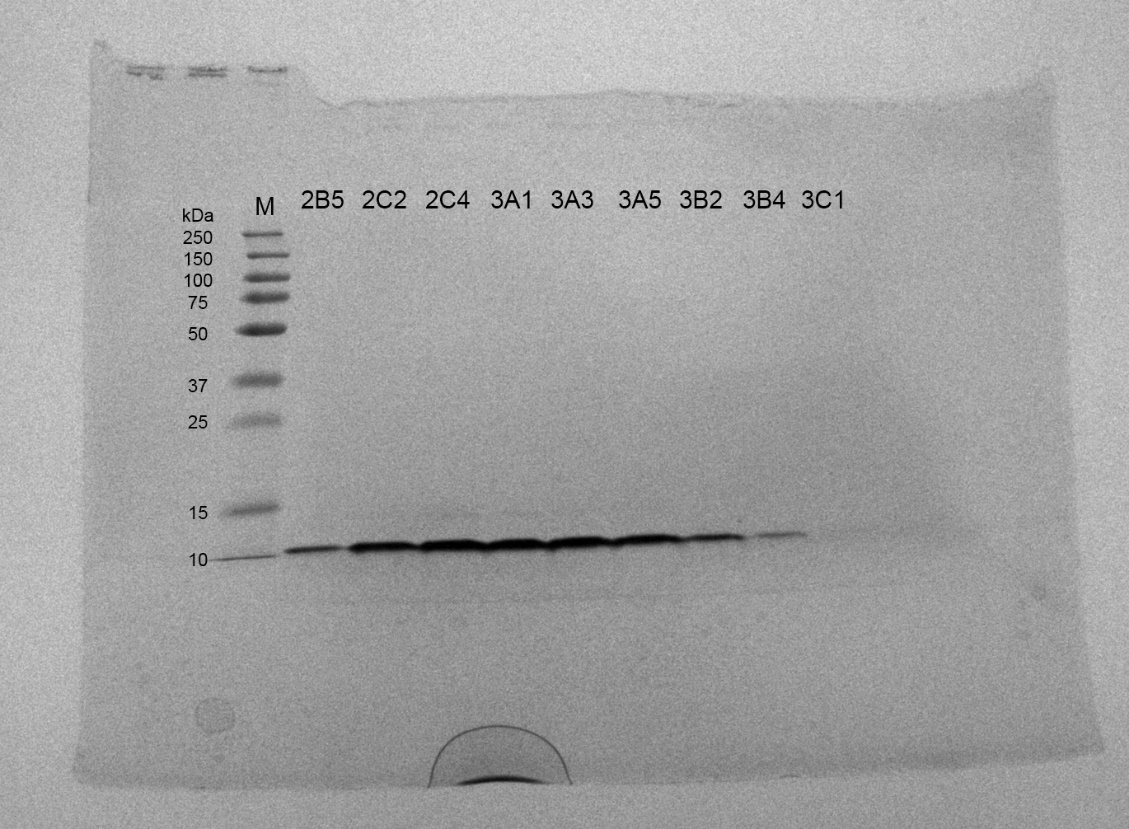


**Full SDS gel image for Supplementary Fig S4**


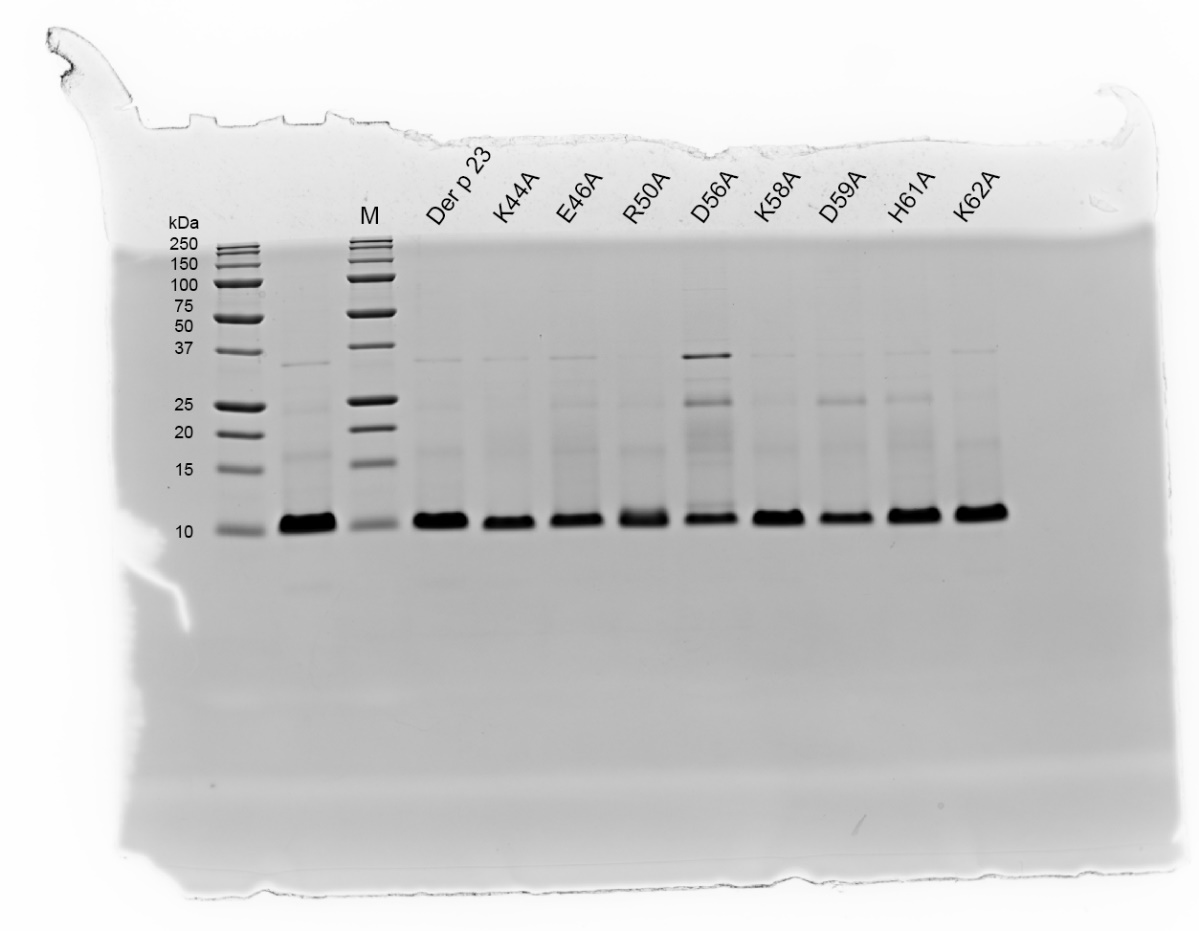


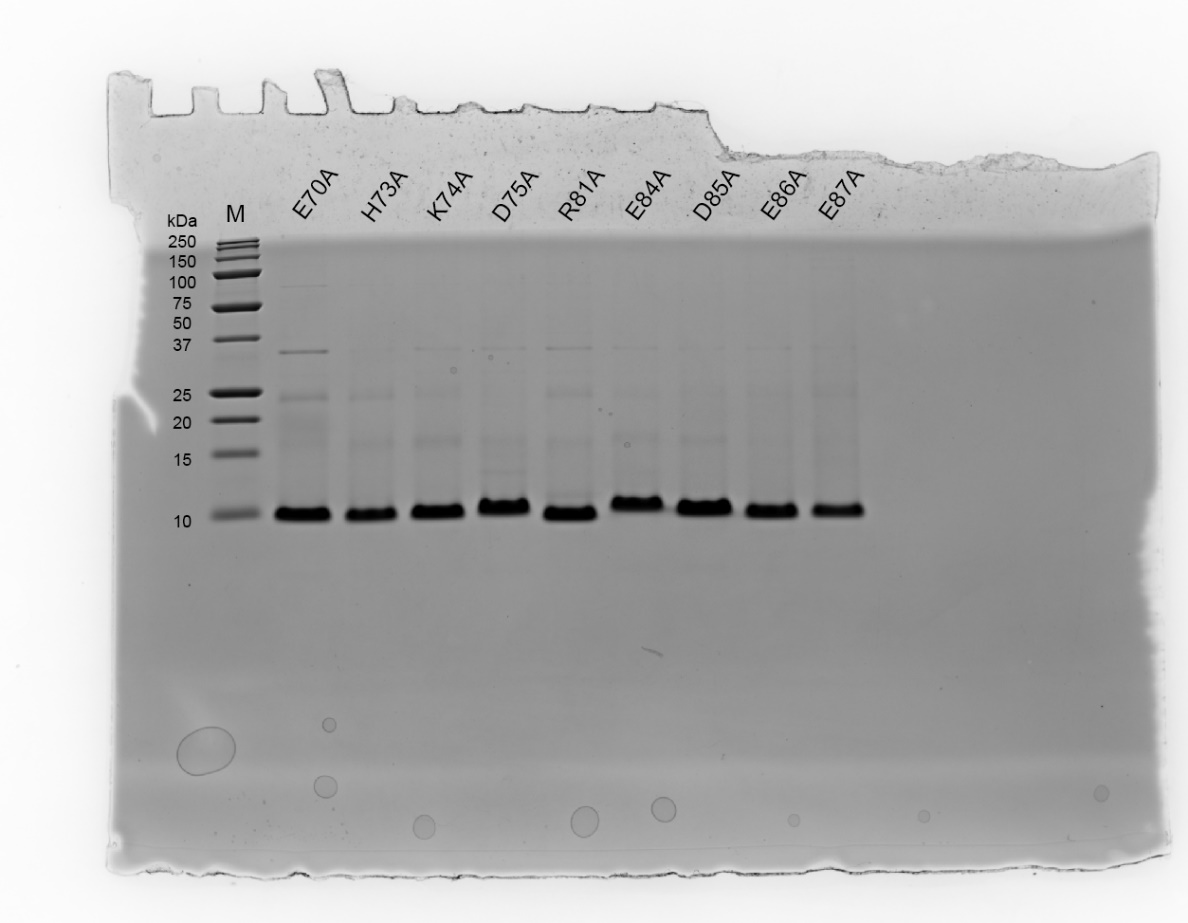

Supplement: Supplementary file 1 — Supplementary Information. [file 41598_2020_79820_MOESM1_ESM.docx]
